# Neuroimaging correlates and biomarker performance of a fully automated plasma p‐tau217/Aβ42 ratio assay in a clinical cohort with Alzheimer's disease

**DOI:** 10.1002/alz.70942

**Published:** 2025-11-24

**Authors:** Yoo Hyun Um, Paul Wynveen, Mark Holland, Kinal Bhatt, Zivjena Vucetic, Brian Engel, Corey Carlson, Andrew Becker, Irene B. Meier, Vaibhav A. Narayan, Sheng‐Min Wang, Dong Woo Kang, Sunghwan Kim, Suhyung Kim, Donghyeon Kim, Yeong Sim Choe, Regina E. Y. Kim, Seunggyun Ha, Hyun Kook Lim

**Affiliations:** ^1^ Department of Psychiatry St. Vincent's Hospital, College of Medicine The Catholic University of Korea Seoul Republic of Korea; ^2^ Beckman Coulter Inc. Research & Development Chaska Minnesota USA; ^3^ Beckman Coulter Inc. Biostatistics Chaska Minnesota USA; ^4^ Beckman Coulter Inc. Medical & Science Affairs Chaska Minnesota USA; ^5^ Davos Alzheimer's Collaborative Geneva Switzerland; ^6^ Department of Psychiatry Yeouido St. Mary's Hospital College of Medicine The Catholic University of Korea Seoul Republic of Korea; ^7^ Department of Psychiatry Seoul St. Mary's Hospital College of Medicine The Catholic University of Korea Seoul Republic of Korea; ^8^ Research Institute Neurophet Inc. Seoul Republic of Korea; ^9^ Division of Nuclear Medicine Department of Radiology Seoul St. Mary's Hospital College of Medicine The Catholic University of Korea Seoul Republic of Korea; ^10^ CMC Institute for Basic Medical Science The Catholic Medical Center of The Catholic University of Korea Seoul Republic of Korea

**Keywords:** Alzheimer's disease, amyloid beta, amyloid positron emission tomography, biomarkers, Braak stage, glial fibrillary acidic protein, magnetic resonance imaging, plasma phosphorylated tau at threonine 217, ratio, tau positron emission tomography

## Abstract

**INTRODUCTION:**

Blood‐based biomarkers offer scalable, non‐invasive tools for Alzheimer's disease (AD) detection. We investigated the performance of plasma biomarkers associated with AD on the automated Beckman Coulter Access DxI 9000 analyzer.

**METHODS:**

This cross‐sectional study included 262 individuals from across the AD continuum. Plasma phosphorylated tau at threonine 217 (p‐tau217), amyloid beta (Aβ)42, and their ratio were measured. Diagnostic accuracy for amyloid positron emission tomography (PET) positivity (Centiloid > 20), using a dual cutoff approach, was assessed via receiver operative characteristic curve. Associations with tau PET (*n* = 76) were also assessed.

**RESULTS:**

The p‐tau217/Aβ42 ratio showed the highest diagnostic accuracy for amyloid PET positivity (area under curve = 0.943) and the smallest indeterminate zone (8.0%). It correlated strongly and consistently with tau PET across Braak stages and with AD‐related cortical atrophy.

**DISCUSSION:**

The p‐tau217/Aβ42 ratio was the most reliable plasma biomarker, closely tracking tau PET. It has potentials for clinical use in diagnosis and treatment monitoring.

**Highlights:**

- This is the first validation of the Beckman Coulter plasma immunoassay.
- The plasma phosphorylated tau at threonine 217 amyloid beta 42 ratio showed the highest accuracy across the full Alzheimer's disease (AD) spectrum.
- Plasma biomarkers correlated with tau positron emission tomography and AD‐related brain atrophy.
- Glial fibrillary acidic protein offered complementary value reflecting astrocytic activation.

## 1 BACKGROUND

Phosphorylated tau at threonine 217 (p‐tau217) has emerged as one of the most reliable and disease‐specific blood‐based biomarkers (BBMs) for Alzheimer's disease (AD). Multiple studies have demonstrated that plasma p‐tau217 shows strong correlations with both amyloid and tau positron emission tomography (PET) signals and distinguishes AD from other neurodegenerative disorders with high accuracy, outperforming earlier p‐tau isoforms such as p‐tau181 and p‐tau231.^1^, ^2^, ^3^, ^4^, ^5^ Recent studies suggest that the combination of p‐tau217 with amyloid beta (Aβ)42, the p‐tau217/Aβ42 ratio, enhances the discriminative performance that effectively captures the core pathological features of AD, which has been validated in multiple independent cohorts.^6^, ^7^ In addition to amyloid and tau biomarkers, glial fibrillary acidic protein (GFAP) serves as a marker of astrocytic activation and neuroinflammation, processes that occur early in AD pathophysiology and may precede overt cognitive decline.^8^

Despite the aforementioned studies supporting the diagnostic potential of the BBMs, evidence from fully automated platforms remain scarce. Fully automated immunoassay platforms address the unmet needs of real‐world clinical settings,^9^ enabling broader adoption across diverse health‐care settings, including primary care and community‐based clinics. Currently, only a few platforms, such as the Beckman Coulter Access system and the Fujirebio Lumipulse G platform offer automated solutions for future clinical application. In this regard, Beckman Coulter's automated immunoassay platform for plasma p‐tau217 and Aβ42 is an important step forward to advance the research and potentially broaden the clinical utility and accessibility of BBMs. Despite this milestone, no peer‐reviewed validation studies have been conducted to assess the analytical and clinical validity of the Beckman Coulter immunoassay.

In addition to establishing the diagnostic accuracy of BBMs in clinical settings, elucidating their associations with in vivo brain pathology via neuroimaging is critical to support their biological validity. Most previous studies have relied on global PET metrics such as whole‐brain standardized uptake value ratio (SUVR),^10^, ^11^ limiting the spatial resolution of BBM–neuroimaging correlations. Moreover, many studies have focused predominantly on symptomatic individuals,^6^, ^12^ limiting the generalizability to asymptomatic or at‐risk individuals. The p‐tau217/Aβ42 ratio has been reported to outperform p‐tau217 alone in discriminative power and to associate with both amyloid and tau PET signals.^13^ However, to date, only one study has demonstrated its association with global amyloid burden and with regional tau PET uptake in the meta‐temporal cortex.^13^ This underscores the need for more comprehensive spatial approaches to clarify the extent to which this ratio captures late‐stage tau pathology across the AD continuum.

**RESEARCH IN CONTEXT**

1. **Systematic review**: We systematically reviewed the current literature on plasma phosphorylated tau at threonine 217 (p‐tau217) and the p‐tau217/amyloid beta (Aβ) 42 ratio as Alzheimer's disease (AD) biomarkers using PubMed and recent conference presentations. Although several assays have shown high diagnostic accuracy, peer‐reviewed validation on the fully automated Beckman Coulter system remains scarce. Few studies have integrated high‐resolution neuroimaging correlations.
2. **Interpretation**: Our findings provide the first independent validation of the Beckman Coulter platform for plasma p‐tau217 and Aβ42. The p‐tau217/Aβ42 ratio demonstrated superior biomarker performance and strong associations with both tau positron emission tomography uptake and structural magnetic resonance imaging changes, even across the full clinical AD spectrum. This highlights the platform's clinical utility and the biomarkers’ potential for disease staging.
3. **Future directions**: Larger, longitudinal studies are needed to validate these biomarkers for tracking disease progression and treatment response. Cross‐platform comparisons will also be essential for clinical harmonization and regulatory decision making.

To address the gaps, we conducted the first independent validation of the Beckman Coulter immunoassays for plasma p‐tau217 and Aβ42 in a clinically characterized cohort. GFAP was also measured on the same Beckman Coulter platform under research use = only conditions. The validation was implemented in a biomarker‐derived, stringently stratified cohort ranging the full AD continuum from cognitively normal individuals to AD dementia. We evaluated the diagnostic performance of the plasma biomarkers and applied a dual‐threshold framework to classify participants regarding amyloid PET positivity. Additionally, we performed multimodal neuroimaging analyses to explore the association of plasma biomarkers with amyloid PET, tau PET, and structural magnetic resonance imaging (MRI).

## 2 MATERIALS AND METHODS

### 2.1 Study design and participants

This cross‐sectional study included 262 participants (≥ 55 years) recruited from the Catholic Aging Brain Imaging Database (CABID) between 2022 and 2024. All participants underwent structural MRI, amyloid PET imaging, and neuropsychiatric evaluations. Neuroimaging and blood sampling were conducted within a maximum interval of 2 years. Individuals were categorized into five diagnostic groups in accordance with the recent revised criteria for AD staging and diagnosis from the Alzheimer's Association workgroup:^14^ Aβ− cognitively unimpaired (Aβ− CU), Aβ+ cognitively unimpaired (Aβ+ CU), Aβ− mild cognitive impairment (MCI), Aβ+ MCI, and AD dementia. Detailed information on the diagnostic criteria, in addition to inclusion and exclusion protocols, can be found in supporting information.

### 2.2 Blood sampling

Blood samples were obtained following a standardized protocol across all participants. Sampling was conducted during routine outpatient visits, primarily in the morning and early afternoon, to minimize potential diurnal variation. Approximately 40 mL of blood was drawn slowly using vacutainer holders and 21‐gauge needles to prevent hemolysis, into ethylenediaminetetraacetic acid–containing tubes. Tubes were gently inverted 8 to 10 times to mix with anticoagulant and centrifuged within 2 hours of collection. Plasma was separated by centrifugation at 1500g for 15 minutes at 23°C without braking, aliquoted carefully to avoid contamination of the buffy coat, briefly vortexed, and stored at –70°C to –80°C until analysis. All samples were processed under identical pre‐analytical conditions at the Catholic Brain Health Center, Department of Psychiatry, Yeouido St. Mary's Hospital, The Catholic University of Korea. Plasma concentrations of p‐tau217 and Aβ42 were measured using the fully automated Beckman Coulter Dxi9000 immunoassay platform.^15^ The p‐tau217/Aβ42 ratio was calculated to evaluate combined amyloid and tau burden. GFAP levels were also measured using the same platform.

### 2.3 Imaging data acquisition and processing

Structural brain imaging and amyloid PET scans were acquired for all participants. MRI acquisition was performed using a 3 Tesla Siemens Skyra scanner (Siemens Healthcare) with a 20‐channel head and neck coil. T1‐weighted anatomical images were collected using a magnetization‐prepared rapid gradient echo (MPRAGE) sequence, with the following imaging parameters: repetition time (TR) = 1860 ms, echo time (TE) = 25.3 ms, flip angle = 9°, field of view (FOV) = 224 × 224 mm, matrix = 256 × 256, 208 axial slices, and slice thickness = 1.0 mm.

Amyloid and tau PET imaging were conducted using a Biograph 40 TruePoint PET scanner (Siemens Medical Solutions). For amyloid PET, participants received an intravenous injection of 185 MBq of [18F]‐flutemetamol, and static PET acquisition was performed 90 minutes post‐injection. For tau PET, 370 MBq of [18F]‐flortaucipir was administered, and imaging began 80 minutes after injection, lasting for 20 minutes. Images were reconstructed with a matrix of 256 × 256 × 175 and voxel dimensions of 1.3364 × 1.3364 × 3 mm^3^. DICOM files were anonymized and converted to NIfTI format using the “dcm2niix” conversion tool.^16^

T1‐weighted MRI data were processed using FreeSurfer version 6.0 (http://surfer.nmr.mgh.harvard.edu), which enabled cortical reconstruction and estimation of cortical thickness based on standard surface‐based algorithms. Amyloid and tau burden was quantified using a deep learning–based automated analysis pipeline (SCALE PET v.0.1.3.1), which computed global SUVR using the pons as the reference region for amyloid PET, and the cerebellum for tau PET.^17^, ^18^ A global SUVR cutoff of 0.62, corresponding to Centiloid (CL)‐defined amyloid positivity,^19^ was applied based on the SCALE PET output. To harmonize tau PET quantification across individuals and sites, centaur values were also calculated based on the CenTauR framework proposed by Dore et al.^20^ To assess voxel‐wise and surface‐level associations between amyloid PET signal intensity and plasma biomarkers, PETSurfer was used for PET–MRI co‐registration and cortical surface rendering (https://surfer.nmr.mgh.harvard.edu/fswiki/PetSurfer).

### 2.4 Cognitive measures and apolipoprotein E genotyping

Originally developed for the standardized clinical and neuropsychological assessment of AD, the Korean version of the Consortium to Establish a Registry for Alzheimer's Disease Assessment Packet (CERAD‑K) has been validated and proven reliable for use in the Korean population.^21^ The CERAD‐K battery included tests such as Verbal Fluency (VF), the 15‐item Boston Naming Test (BNT), Mini‐Mental State Examination Korean version (MMSE‐K),^22^ Word List Memory (WLM), Word List Recall (WLR), Word List Recognition (WLRc), Constructional Praxis (CP), and Constructional Recall (CR). A neuropsychologist reviewed the results to determine the presence of cognitive impairment. Each test was scored according to established criteria: VF measured the number of animal names generated in 1 minute, while BNT had a maximum score of 15 points. The MMSE‐K ranged from 0 to 30 points, WLM from 0 to 30 points, WLR and WLRc from 0 to 10 points each, and CP and CR from 0 to 11 points each. Two CERAD‐K total scores, designated as total scores (TS)‐I and TS‐II, were computed.^23^ TS‐I is produced by aggregating the scores derived from six assessments, which encompass the VF, BNT, WLM, CP, WLR, and WLRc.^23^ TS‐II is defined by the addition of CR to TS‐I.^23^ Apolipoprotein E (*APOE*) genotyping (as detailed in supporting information) was conducted for every participant.

### 2.5 Statistical analyses

Descriptive statistics were used to summarize demographic and clinical characteristics across the five diagnostic groups. Group differences in continuous variables were evaluated using one‐way analysis of variance (ANOVA), while categorical variables were compared using chi‐squared tests. All tests were two tailed, and statistical significance was set at *P* < 0.05. To compare plasma biomarker levels across diagnostic groups, analysis of covariance (ANCOVA) was conducted with adjustment for age and sex. Post hoc pairwise comparisons were performed using Bonferroni correction to identify specific group‐level differences. The influence of *APOE* ε4 allele carrier status on plasma biomarker concentrations was examined using independent‐samples *t* tests. Each biomarker (p‐tau217, Aβ42, p‐tau217/Aβ42 ratio, and GFAP) was compared between *APOE* ε4 carriers and non‐carriers.

Receiver operating characteristic (ROC) curve analysis was implemented to evaluate the diagnostic performance of each plasma biomarker for predicting amyloid PET positivity (defined as CL > 20, with reference to a previous study^24^). ROC curves were generated by training each biomarker as the independent variable and amyloid status (positive vs. negative) as the dependent variable. The area under the curve (AUC) and corresponding 95% confidence intervals (CIs) were calculated. ROC analyses were performed in a subsample of 238 individuals for whom CL derived from SCALE PET were available. To assess the additional diagnostic value of genetic predisposition, *APOE* ε4 carrier status was integrated as a covariate in addition to each biomarker as the independent variable in logistic regression models. Comparative ROC analyses with and without *APOE* ε4 inclusion were conducted for each biomarker. Additionally, subgroup analyses were performed separately for cognitively unimpaired (Aβ+ CU and Aβ+ CU) and cognitively impaired (MCI + AD dementia) individuals.

To estimate classification performance, a two‐cutoff approach (using upper and lower cutoffs) was used in accordance with the Alzheimer's Association appropriate use recommendations for AD blood biomarkers.^25^ Dual cutoff thresholds for p‐tau217 and the p‐tau217/Aβ42 ratio were derived using a bootstrap‐based method (1000 iterations). Based on the lower and upper thresholds, an intermediate zone was defined. The goal was to minimize the proportion of individuals falling into this indeterminate range while optimizing the following performance metrics: positive predictive value (PPV ≥ 90%), negative predictive value (NPV ≥ 90%), positive likelihood ratio (PLR > 5.0), and negative likelihood ratio (NLR < 0.1).^6^, ^26^ All metrics were calculated along with their 95% CIs.

Structural MRI and amyloid PET imaging were available for all 262 participants, and a subset of 76 participants additionally underwent tau PET imaging with [18F]‐flortaucipir. Vertex‐wise associations between plasma biomarkers and neuroimaging metrics—including amyloid PET uptake, tau PET uptake, cortical volume, and cortical thickness—were analyzed using surface‐based general linear models implemented in FreeSurfer. Analyses were adjusted for age, sex, and total intracranial volume (TIV). A smoothing kernel of 5 mm full width at half maximum (FWHM) was applied for PET data and 10 mm FWHM for structural MRI. Statistical significance was determined using a cluster‐wise correction for multiple comparisons based on Monte Carlo simulations (10,000 iterations), with a cluster‐wise *P* value threshold of <0.05. Partial correlations between plasma biomarker levels and regional tau PET measures were computed after adjusting for age and sex via a residual‐based linear regression approach. To further explore cognitive stage–specific associations, subgroup analyses were conducted on CU and cognitively impaired participants. Partial correlation analyses were independently conducted within each subgroup. Regional tau PET SUVRs were categorized according to Braak stage–related cortical topography, based on predefined variable groupings derived from the CenTauR framework.^20^ The mapping of region variables to approximate Braak stages is summarized in supporting information. Statistical software details are elaborated in supporting information.

## 3 RESULTS

### 3.1 Demographic and clinical characteristics of the participants

A total of 262 subjects were included in the analyses and demographic and clinical characteristics of the participants are summarized in Table 1. They were divided into five diagnostic groups according to their PET status and CERAD‐K results: Aβ− CU (*N* = 77), Aβ+ CU (*N* = 24), Aβ− MCI (*N* = 62), Aβ+ MCI (*N* = 78), and AD dementia (*N* = 21). There was a significant difference in age between groups (*P*  <  0.001). There were no significant group differences in sex (*P*  =  0.459) or years of education (*P*  =  0.482). Higher global SUVR was observed in groups with more advanced cognitive impairment, with Aβ+ CU (0.70  ±  0.07), Aβ+ MCI (0.73  ±  0.09), and AD dementia (0.77  ±  0.08), compared to Aβ− CU (0.47  ±  0.07) and Aβ− MCI (0.47  ±  0.08; *P*  <  0.001). Both CERAD‐K TS‐I and TS‐II differed significantly across groups (*P*  <  0.001), with the lowest scores observed in the AD dementia group.

**TABLE 1:** Demographic and clinical characteristics by diagnostic group (*N* = 262).

	Aβ− CU (*N* = 77)	Aβ+ CU (*N* = 24)	Aβ− MCI (*N* = 62)	Aβ+ MCI (*N* = 78)	AD dementia (*N* = 21)	*P* value
Age (years)	72.22 ± 8.03	77.96 ± 6.21	77.79 ± 7.38	74.28 ± 7.64	70.24 ± 10.83	<0.001
Sex (proportion female, %)	72.73%	83.33%	67.74%	65.38%	76.19%	0.459
Education (years)	11.25 ± 4.60	9.29 ± 4.59	10.69 ± 5.00	10.99 ± 5.32	10.10 ± 4.25	0.482
Global SUVR	0.47 ± 0.07	0.70 ± 0.07	0.47 ± 0.08	0.73 ± 0.09	0.77 ± 0.08	<0.001
Total CERAD‐K score 1	70.90 ± 11.22	66.58 ± 11.92	53.18 ± 9.94	51.00 ± 11.18	30.52 ± 13.13	<0.001
Total CERAD‐K score 2	78.01 ± 13.28	72.21 ± 15.00	56.37 ± 10.82	53.47 ± 12.75	30.71 ± 13.24	<0.001

Notes: All values are presented as mean ± standard deviation unless otherwise specified. Total CERAD‐K score 1, composite score based on six core CERAD‐K subtests; Total CERAD‐K score 2, Total CERAD‐K score 1 plus constructional recall.

Abbreviations: Aβ, amyloid beta; AD, Alzheimer's disease; CERAD, Consortium to Establish a Registry for Alzheimer's Disease; CU, cognitively unimpaired; GFAP, glial fibrillary acidic protein; MCI, mild cognitive impairment; p‐tau217, phosphorylated tau at threonine 217; SUVR, standardized uptake value ratio.

### 3.2 Group‐wise comparisons of plasma biomarker levels

**FIGURE 1:**
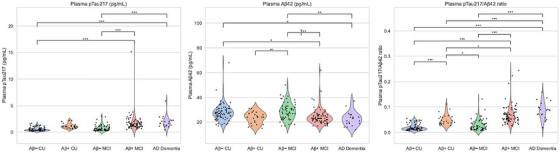
Group‐wise distribution of plasma biomarkers across diagnostic categories (*N* = 262). Aβ, amyloid beta; AD, Alzheimer's disease; CU, cognitively unimpaired; MCI, mild cognitive impairment; p‐tau217, phosphorylated tau at threonine 217

Age‐ and sex‐adjusted ANCOVA revealed significant differences across groups in all plasma biomarkers (ANCOVA, all *P* <  0.001; Figure 1 and Table S1 in supporting information). Mean p‐tau217 levels were significantly higher in Aβ+ CU (1.15  ±  0.46 pg/mL), Aβ+ MCI (1.75  ±  1.69), and AD dementia (1.95  ±  1.11) compared to Aβ− CU (0.55  ±  0.36; *P*  <  0.001). Aβ42 concentrations were significantly lower in Aβ+ CU (23.54  ±  4.63) and AD dementia (22.62  ±  5.56) compared to Aβ− CU (27.64  ±  6.91; *P*  <  0.001). The p‐tau217/Aβ42 ratio was significantly higher in subjects with AD compared to Aβ− CU, with AD dementia showing the highest value (0.086  ±  0.034, *P*  <  0.001). GFAP levels were also significantly higher in Aβ+ CU (20.67 ± 10.63), Aβ+ MCI (21.55 ± 9.53), and AD dementia (22.81 ± 11.04) than in Aβ− CU (12.30  ±  7.36, *P*  <  0.001). Among the plasma biomarkers, the p‐tau217/Aβ42 ratio exhibited the most robust group‐wise discrimination (Figure 1, Table S1). Post hoc pairwise comparisons revealed statistically significant discrimination between Aβ− CU and subjects in Aβ+ CU, Aβ+ MCI, and AD dementia. Moreover, it significantly differentiated intermediate stages such as Aβ− MCI versus Aβ+ MCI and Aβ+ CU versus Aβ+ MCI. Although group‐wise comparisons were performed across the full AD continuum, results from the smaller subgroups should be interpreted as exploratory.

### 3.3 Plasma biomarker profiles based on *APOE* ε4 genetic status

**FIGURE 2:**
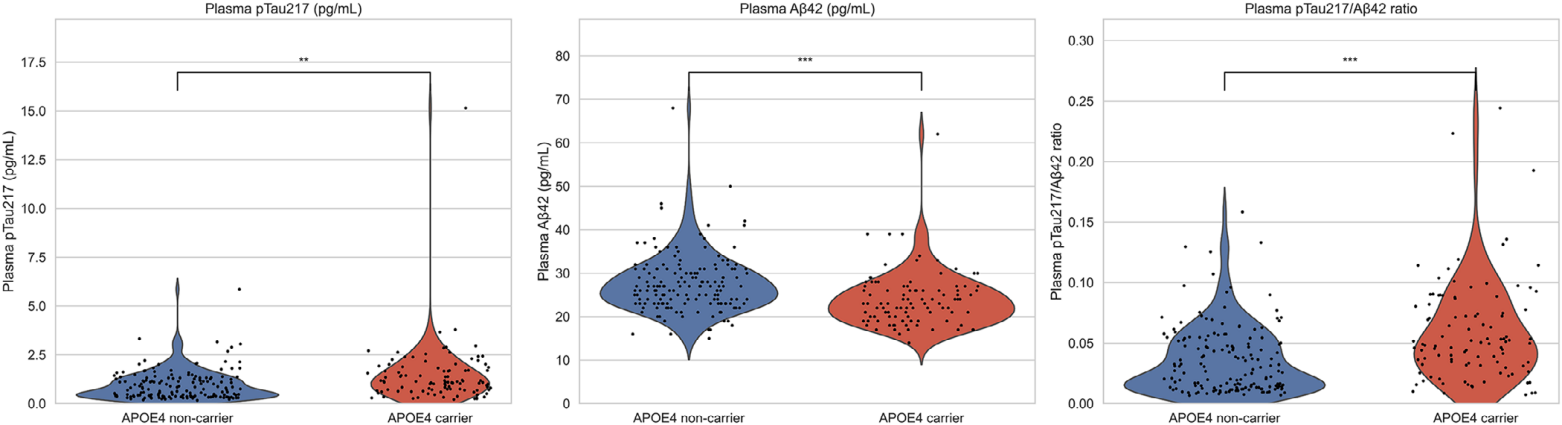
Comparison of biomarker levels between *APOE* ε4 carriers and non‐carriers (*N* = 262). Aβ, amyloid beta; *APOE*, apolipoprotein E; p‐tau217, phosphorylated tau at threonine 217

*APOE* ε4 carriers (*N* = 102) showed significantly elevated p‐tau217 (1.43  ±  1.58 vs. 0.94  ±  0.76 pg/mL, *P*  =  0.005) and lower Aβ42 levels (23.87  ±  6.31 vs. 27.61  ±  6.71 pg/mL, *P*  <  0.001) compared to non‐carriers (*N* = 160). The p‐tau217/Aβ42 ratio was also significantly higher in carriers (0.059  ±  0.042 vs. 0.036  ±  0.028, *P*  <  0.001). Differences in GFAP levels were not statistically significant (*P*  =  0.090; Figure 2 and Table S2 in supporting information).

### 3.4 Discriminative performance of plasma biomarkers

**FIGURE 3:**
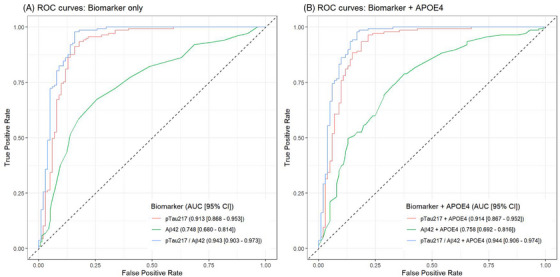
ROC curve comparison for (A) biomarker‐only versus (B) biomarker+ *APOE* ε4 models (*N* = 238). CL value > 20 was used to define amyloid positivity. Aβ, amyloid beta; *APOE*, apolipoprotein E; AUC, area under the curve; CI, confidence interval; CL, Centiloid; p‐tau217, phosphorylated tau at threonine 217; ROC, receiver operating characteristic

ROC analysis for predicting amyloid PET positivity (defined as CL  >  20) showed that the p‐tau217/Aβ42 ratio had the highest discriminative performance (AUC  =  0.943), followed by p‐tau217 (AUC  =  0.913), GFAP (AUC  =  0.813), and Aβ42 (AUC = 0.748; Table S3 in supporting information and Figure 3A). When *APOE* ε4 was inserted in the model, it did not affect the discriminative performance for p‐tau217/Aβ42 ratio (AUC = 0.944), p‐tau217 (AUC  =  0.914), GFAP (AUC  =  0.842), and Aβ42 (AUC = 0.758; Table S3, Figure 3B). In CU individuals, the p‐tau217/Aβ42 ratio demonstrated an AUC of 0.945, still maintaining high diagnostic performance, which was followed by p‐tau217 (AUC = 0.907), GFAP (AUC = 0.836), and Aβ42 (AUC = 0.718; Table S4 and Figure S1 in supporting information). In cognitively impaired individuals, p‐tau217/Aβ42 ratio still exhibited the highest discriminative performance with an AUC of 0.907, followed by p‐tau217 (AUC  =  0.879), GFAP (AUC  =  0.737), and Aβ42 (AUC = 0.785; Table S5 and Figure S2 in supporting information). In subgroup analyses of both CU and cognitively impaired subjects, insertion of *APOE* ε4 in the model did not affect diagnostic performance of p‐tau217/Aβ42 ratio and p‐tau217, but it slightly improved the performance of Aβ42 and GFAP (Figures S1, S2).

### 3.5 Subject classification metrics of p‐tau217 and p‐tau217/Aβ42 ratio using dual cutoffs

Bootstrap‐derived cutoffs for p‐tau217 and the p‐tau217/Aβ42 ratio identified intermediate zones of 13.4% and 8.0% of the participants, respectively. The p‐tau217/Aβ42 ratio showed high diagnostic utility with a PPV of 91.2% (95% confidence interval [CI]: 84.9–95.0) and NPV of 90.4% (95% CI: 82.8–94.9). The corresponding PLR was 7.64 (95% CI: 4.35–13.41), and NLR was 0.08 (95% CI: 0.04–0.15; Table 2).

**TABLE 2:** Bootstrap‐based classification performance metrics for p‐tau217 and p‐tau217/Aβ42 ratio (*N* = 238).

	N	Lower cutoff	Upper cutoff	Intermediate zone %	PPV%	NPV%	PLR	NLR	PLR 95% CI	NLR 95% CI	PPV % 95% CI	NPV % 95% CI	Intermediate PLR	Intermediate PLR 95% CI
p‐tau217	238	0.712	1.048	13.4	90.4	90.2	6.90	0.08	3.92–12.16	0.04–0.15	83.5–94.5	82.4–94.8	2.63	1.16–5.97
p‐tau217/Aβ42 ratio	238	0.0297	0.0374	8.0	91.2	90.4	7.64	0.08	4.35–13.41	0.04–0.15	84.9–95.0	82.8–94.9	2.06	0.76–5.64

Notes: Binary classification performance was evaluated using bootstrapped analysis to determine optimal lower and upper cutoffs for each biomarker (p‐tau217, p‐tau217/Aβ42 ratio). The intermediate zone was defined as values falling between these thresholds. PPV, NPV, PLR, NLR, and their 95% CIs are reported. Intermediate PLR indicates the likelihood ratio within the intermediate zone. Percentages are shown for the proportion of subjects falling into the intermediate zone.

Abbreviations: Aβ, amyloid beta; CI, confidence interval; NLR, negative likelihood ratio; NPA, negative percent agreement; NPV, negative predictive value; PLR, positive likelihood ratio; PPA, positive percent agreement; PPV, positive predictive value; p‐tau217, phosphorylated tau at threonine 217.

### 3.6 Neuroimaging correlates of plasma biomarkers

**FIGURE 4:**
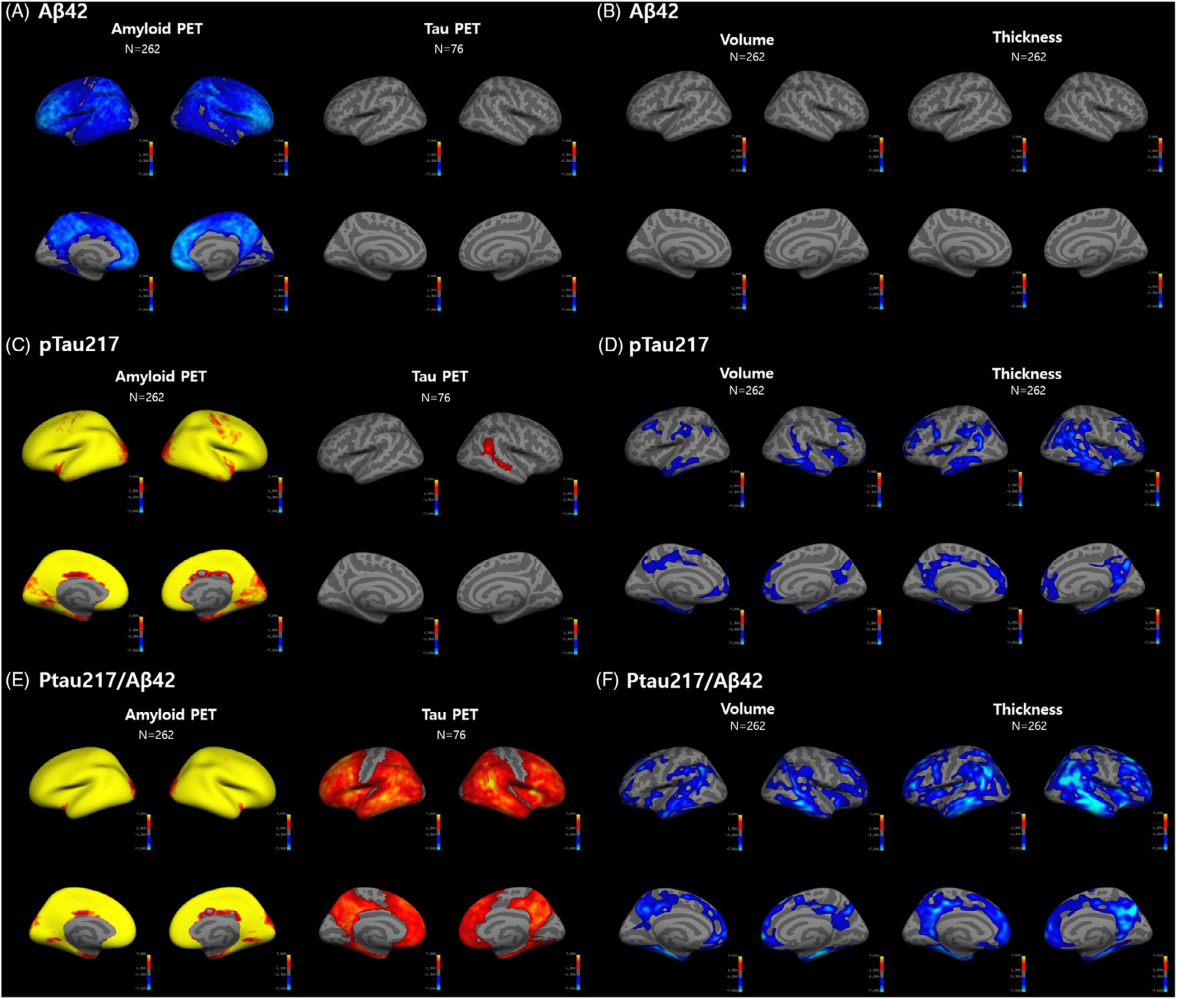
Spatial associations of plasma biomarkers with neuroimaging measures. Cortical surface maps illustrate the spatial associations between plasma biomarkers and neuroimaging markers across the Alzheimer's disease continuum. Each row corresponds to one plasma biomarker: (A–B) Aβ42, (C–D) p‐tau217, and (E–F) p‐tau217/Aβ42. For each biomarker, the left panel shows associations with PET measures (amyloid and tau PET), and the right panel shows associations with structural MRI measures (cortical volume and thickness). Warm colors (red–yellow) indicate positive associations, and cool colors (blue) indicate negative associations. All associations are adjusted for age, sex, and TICV, and visualized using a *t* statistic threshold of |*t*| > 1.3. Aβ, amyloid beta; MRI, magnetic resonance imaging; PET, positron emission tomography; p‐tau217, phosphorylated tau at threonine 217; TIV, total intracranial volume.

Neuroimaging analyses revealed significant associations between plasma biomarkers and amyloid PET, tau PET, and structural MRI measures (Figure 4 and Tables S6–S9 in supporting information). Lower plasma Aβ42 was significantly associated with higher amyloid PET signal in the left rostral middle frontal and right medial orbitofrontal gyri (Figure 4A, Table S6). No clusters survived for the association between plasma Aβ42 and structural MRI measures (Figure 4B). Higher plasma p‐tau217 levels were associated with increased amyloid PET uptake in left precuneus and right supramarginal gyrus and were positively correlated with tau PET uptake in the right inferior parietal gyrus (Figure 4C, Table S7). Decreased volumes in association with higher plasma p‐tau217 levels were observed in multiple cortical regions including the left superior frontal, posterior cingulate, supramarginal, caudal middle frontal, inferior parietal gyri, right fusiform, medial orbitofrontal gyri and precuneus (Figure 4D, Table S7). Cortical thinning of the left precentral, parahippocampal, superior frontal gyri, inferior temporal, entorhinal, posterior cingulate gyri, right lateral orbitofrontal, pars triangularis, and parahippocampal gyri was observed with increased plasma p‐tau217 levels (Figure 4D, Table S7). Increased amyloid PET uptake in bilateral precuneus and increased tau PET uptake in left middle temporal and right inferior parietal gyri were associated with higher p‐tau217/Aβ42 ratio (Figure 4E, Table S8). A higher p‐tau217/Aβ42 ratio was associated with decreased volumes in the left entorhinal orbitofrontal and caudal middle frontal gyri while cortical thinning was observed in the left banks of the superior temporal and superior frontal gyri (Figure 4F, Table S8). Higher plasma GFAP levels were associated with increased amyloid PET uptake in bilateral precuneus and tau PET uptake in the left parahippocampal and right insula, and entorhinal gyri (Table S9). Decreased volumes in bilateral fusiform, precuneus, left superior frontal, rostral middle frontal, right medial orbitofrontal, caudal middle frontal, and supramarginal gyri were noted with increased plasma GFAP levels (Table S9). Cortical thinning of the left fusiform and superior frontal gyri was also observed (Table S9).

### 3.7 Associations between plasma biomarkers and regional tau PET uptake

**FIGURE 5:**
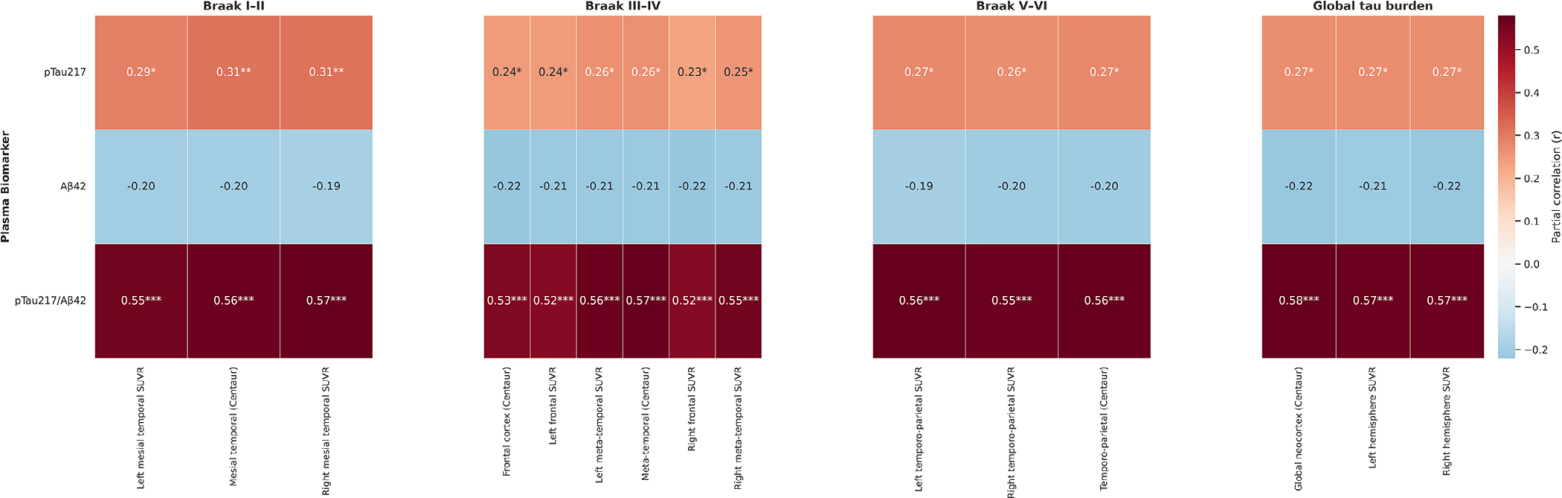
Partial correlations between plasma biomarkers and tau PET SUVRs, stratified by Braak stage (*N* = 76). Each heatmap represents partial correlation coefficients (*r*) between four plasma biomarkers and tau PET SUVR values in regions approximating Braak stages: (1) Braak I–II (transentorhinal/mesial temporal), (2) Braak III–IV (limbic/meta‐temporal and associative neocortex), (3) Braak V–VI (isocortical/neocortical), and (4) global tau burden. All correlations are adjusted for age and sex. Warm colors indicate positive correlations, and cool colors indicate negative correlations. Asterisks denote statistical significance: *P < 0.05, **P < 0.01, ***P < 0. 001. Aβ, amyloid beta; PET, positron emission tomography; p‐tau217, phosphorylated tau at threonine 217; SUVR, standardized uptake value ratio

We examined age‐ and sex‐adjusted partial correlations between plasma biomarkers and regional tau PET SUVRs across 15 predefined regions categorized by Braak stage–defined cortical topography. Results from the full cohort (*n* = 76) are illustrated in a Braak stage–stratified heatmap (Figure 5) and detailed in Table S10 in supporting information.

Across the cohort, the p‐tau217/Aβ42 ratio and GFAP exhibited the strongest and most consistent positive correlations with tau PET SUVRs. The p‐tau217/Aβ42 ratio showed significant associations with both early‐ and late‐stage regions, including the mesial temporal cortex (*r* = 0.56, *P* < 0.0001) and temporo‐parietal cortex (*r *= 0.56, *P* < 0.0001). Similarly, GFAP correlated with tau uptake across Braak stages, with higher values in regions such as the mesial temporal cortex (*r* = 0.46, *P* < 0.0001), frontal cortex (*r* = 0.35, *P* = 0.0002), and temporo‐parietal cortex (*r* = 0.39, *P* < 0.0001). In contrast, p‐tau217 showed moderate but significant correlations (*r* = 0.23–0.31, *P* = 0.007–0.043), while Aβ42 consistently demonstrated non‐significant correlations (e.g., *r *= –0.18 to –0.22, *P* = 0.08–0.15).

Subgroup analyses revealed differential patterns (Tables S11–S12 and Figures S3–S4 in supporting information). In the CU group (*N* = 24), p‐tau217/Aβ42 ratio and p‐tau217 showed strong correlations in Braak III to IV and V to VI regions. For example, the meta‐temporal region showed *r* = 0.69 (*P* < 0.001), and temporo‐parietal cortex *r* = 0.41 (*P* < 0.049) for the ratio. In early‐stage Braak I and II regions, p‐tau217 was significantly associated with tau deposition in early‐stage Braak I and II regions (mesial temporal CenTaur; *r* = 0.57, *P* = 0.004), and the ratio also remained significant (*r* = 0.56, *P* = 0.004). Aβ42 in the CU group showed significant inverse correlations across regions, including *r* = –0.45 (*P* = 0.028) in mesial temporal, and *r *= –0.52 (*P* = 0.009) in Braak III and IV. GFAP showed regionally restricted significance, with modest correlations in Braak III and IV (e.g., left meta‐temporal *r* = 0.46, *P* = 0.02; meta‐temporal CenTaur *r* = 0.45, *P* = 0.027), but did not reach significance in global tau burden regions.

In contrast, in the cognitively impaired group (*N* = 52), the p‐tau217/Aβ42 ratio again showed robust correlations across all Braak stages (*r* = 0.42–0.47, *P* = 0.001–0.003). GFAP remained moderately associated across stages (e.g., *r* = 0.38–0.41 in Braak I–II, *P* = 0.005–0.002; *r *= 0.32–0.37 in global tau burden, *P* = 0.006–0.023). However, p‐tau217 failed to show statistically significant associations in the cognitively impaired (*r* = 0.11–0.19, *P* = 0.183–0.455). Aβ42 again showed non‐significant correlations (*r* = –0.19 to –0.12, *P* = 0.173–0.391).

## 4 DISCUSSION

The present study demonstrated the first comprehensive validation of the Beckman Coulter plasma‐based AD biomarker assays. To our knowledge, this investigation represents the inaugural effort to elucidate the clinical relevance of the plasma‐based AD assay using the Beckman Coulter platform, particularly in relation to plasma p‐tau217 and the p‐tau217/Aβ42 ratio, both of which effectively capture essential pathological signatures of AD and demonstrate a robust correlation with established neuroimaging biomarkers. These findings suggest the platform's potentials for utility as a clinically validated, non‐invasive diagnostic aid across the AD continuum.

In this study, the Beckman Coulter plasma assays effectively distinguished individuals across the AD continuum. Among all biomarkers, the p‐tau217/Aβ42 ratio showed the strongest group differentiation, with markedly higher levels in Aβ‐positive individuals. p‐tau217 alone also demonstrated separation, but the ratio provided superior discrimination, consistent with findings from the ALZAN cohort.^6^ Bonferroni‐corrected post hoc tests confirmed significant differences in the p‐tau217/Aβ42 ratio across all cognitive stages and by Aβ positivity status, supporting its diagnostic robustness. In line with previous studies,^27^, ^28^ *APOE* ε4 carriers exhibited higher plasma p‐tau217 and p‐tau217/Aβ42 ratios and lower Aβ42 levels than non‐carriers, reflecting greater pathological burden. Accordingly, the influence of *APOE* ε4 on diagnostic accuracy was further examined in ROC analyses.

In our ROC curve analyses, the plasma p‐tau217/Aβ42 ratio achieved the highest discriminative performance for amyloid PET positivity (AUC = 0.943), outperforming individual biomarkers including p‐tau217 (AUC = 0.913), GFAP (AUC = 0.813), and Aβ42 (AUC = 0.748). These findings are consistent with prior studies reporting the superiority of p‐tau217/Aβ42 ratio over a single measure of p‐tau217, regardless of the measurement by immunoassay or mass spectrometry.^6^, ^13^ Notably, the recent PrecivityAD2 test (C2N Diagnostics), which measures %p‐tau217 and the Aβ42/40 ratio via mass spectrometry, demonstrated an AUC of 0.94 for detecting amyloid PET positivity (CL > 25) showing strong concordance with PET findings.^29^ This comparable performance underscores the analytical validity of the Beckman Coulter plasma assay. Moreover, this high performance of the ratio was maintained in both cognitively unimpaired (AUC = 0.945) and cognitively impaired (AUC = 0.907) subgroups. The robust performance of plasma p‐tau217/Aβ42 in the cognitively unimpaired group (AUC > 0.94) supports its use in preclinical detection and participant screening for therapeutic trials.^30^ This is particularly relevant in light of recent anti‐amyloid monoclonal antibody trials, which require cost‐effective, scalable screening tools for participant enrichment.^31^

The plasma p‐tau217 alone demonstrated strong diagnostic performance in our cohort (AUC = 0.913), comparable to leading research assays. In a recent head‐to‐head comparison, plasma p‐tau217 immunoassays from Lilly, ALZpath, and Janssen showed AUCs of 0.94, 0.93, and 0.91, respectively.^1^ Moreover, The Lumipulse p‐tau217 assay also achieved AUCs of 0.93 to 0.96 across five independent cohorts.^12^ In CU participants, our assay maintained an AUC of 0.907, consistent with previous reports of p‐tau217 performance in Aβ+ CU.^32^ Although GFAP and Aβ42 showed lower and less consistent performance, they may provide complementary diagnostic value. Adding *APOE* ε4 status to the models had minimal effect on p‐tau217 and p‐tau217/Aβ42 ratio performance—consistent with prior studies,^30^, ^31^ but modestly improved GFAP and Aβ42 classification. A two‐cutoff approach identified smaller intermediate zones for the p‐tau217/Aβ42 ratio (8.0%) compared to p‐tau217 (13.4%), with high PPV and NPV (>90%). These results are in line with immunoassay‐based studies and Lumipulse data reporting reduced indeterminate classifications for the ratio.^6^, ^12^

Beyond their diagnostic capacity, the plasma biomarkers analyzed in the current investigation exhibited meaningful correlations with established neuroimaging measures. Lower Aβ42 levels were associated with increased amyloid PET uptake in medial and lateral frontal cortices, while higher p‐tau217 and p‐tau217/Aβ42 levels were correlated with amyloid burden in the precuneus, a region known to accumulate Aβ in preclinical stages.^33^ Tau PET correlations showed that elevated plasma p‐tau217 and p‐tau217/Aβ42 ratio were associated with greater tau PET uptake in inferior parietal and temporal regions. Tau PET signals in inferior parietal and temporal regions correspond to both limbic (Braak II–IV) and neocortical (Braak V–VI) stages of tau deposition.^34^ These spatial patterns may be contributed to the full clinical and pathological spectrum of our cohort, which included Aβ− CU to AD dementia. Although prior studies have emphasized the association between p‐tau217 with early amyloid pathology, our findings suggest that p‐tau217–based plasma biomarkers may also reflect tau burden in later Braak stage regions, emphasizing their utility not only for early detection of AD but also for staging and disease monitoring across the AD continuum, as suggested in previous studies.^35^, ^36^ As for GFAP, like p‐tau217 and p‐tau217/Aβ42 ratio, there was a positive correlation with amyloid PET uptake in the precuneus, and tau PET uptake extending to limbic stages of tau deposition. The aforementioned association may reflect astrocytic response to early cortical amyloid deposition^37^ and glial activation secondary to tau‐related neurodegeneration,^38^ supporting its role as a marker of astrocytic response across the pathologic cascade of AD.

Structural MRI analyses also revealed the pathophysiological significance of plasma biomarkers. Elevated levels of p‐tau217, p‐tau217/Aβ42, and GFAP were associated with reduced cortical volume and thickness in AD‐vulnerable regions, including the entorhinal cortex, medial orbitofrontal cortex, fusiform gyrus, and posterior cingulate cortex.^39^ Our results are in line with a recent systematic review showing inverse correlations between plasma p‐tau217 and temporal gray matter volume and thickness.^40^ The p‐tau217/Aβ42 ratio showed the most widespread and robust associations with both volumetric and cortical thickness metrics, suggesting it may best reflect downstream neurodegeneration involved. GFAP also showed significant correlations with frontotemporal–parietal cortical atrophy, which aligns with the previous study in which GFAP levels were negatively associated with hippocampal atrophy and lower cortical thickness in temporal and parietal regions in the AD continuum.^40^

When tau PET uptake was aggregated to reflect cortical regions according to Braak stages, distinct patterns of association with plasma biomarkers were evident. Across the entire cohort, the p‐tau217/Aβ42 ratio demonstrated robust and statistically significant positive correlations across all Braak stages, supporting its role as a sensitive and stage‐independent indicator of tau pathology. GFAP, reflecting astrocytic activation, also showed widespread associations from Braak I and II through Braak V and VI, suggesting that astrocytic responses accompany tau accumulation throughout the disease continuum. These findings were consistent in both CU and cognitively impaired groups, although the strength of associations varied by clinical stage. In the CU group, p‐tau217 and p‐tau217/Aβ42 showed especially strong correlations in early and intermediate Braak stages, while Aβ42 displayed significant inverse correlations, indicating early‐phase sensitivity. In contrast, the cognitively impaired group demonstrated more moderate but consistent correlations between the p‐tau217/Aβ42 ratio and tau PET SUVRs, whereas p‐tau217 alone did not reach statistical significance. Importantly, GFAP showed meaningful correlations across Braak stages in both cognitively unimpaired and cognitively impaired, with stronger associations in the latter, highlighting its potential utility as a stage‐transcending marker of neuroinflammation. Consistent with previous *post mortem* and longitudinal studies, elevated GFAP was associated with tau pathology independent of Aβ deposition,^41^ and faster cognitive decline in Aβ+ CU subjects,^42^ supporting a close interplay between astrocytic activation and tau accumulation in driving cognitive deterioration.

This study has several limitations. First, its cross‐sectional design precludes assessment of longitudinal biomarker trajectories or prediction of clinical progression. Second, the sample size for tau PET was smaller than for amyloid PET, which may have limited statistical power. Third, although the cohort covered the full clinical spectrum, certain subgroups (e.g., Aβ+ CU, AD dementia) were modest in size. Thus, analyses were exploratory and aimed at evaluating the assay's biological validity across multimodal markers. Findings should be interpreted as preliminary evidence rather than definitive clinical thresholds. Fourth, the proportion of female participants was relatively high, reflecting the greater prevalence of AD and longer life expectancy among women; however, sex‐adjusted analyses yielded consistent results. Finally, while the Beckman Coulter immunoassay showed strong performance and biological relevance, direct comparison to other platforms was not performed. Future studies should include larger, longitudinal, and more diverse cohorts and benchmark across platforms to confirm clinical applicability and support assay harmonization.

Taken together, our findings demonstrate that plasma biomarkers reflect complementary aspects of AD pathology. The p‐tau217/Aβ42 ratio showed the strongest and most consistent associations across disease stages, highlighting its robustness. GFAP emerged as a marker of astrocytic activation linked to tau pathology, while p‐tau217 alone was informative but showed slightly weaker correlation. These stage‐ and phenotype‐specific patterns underscore the importance of multiplex plasma biomarker profiling to enhance tau pathology tracking across the AD continuum. However, the number of participants in the Aβ+ CU subgroup (*n* = 24) and AD dementia (*N* = 21) was modest, and the corresponding analyses should therefore be interpreted as exploratory rather than conclusive.

This study provides the first independent validation of the Beckman Coulter plasma immunoassays for p‐tau217 and Aβ42 in an East Asian cohort, offering both cultural and genetic relevance to global AD diagnostic efforts.^43^ Rigorous clinical and biomarker stratification across the full disease spectrum and high‐resolution neuroimaging analyses enabled precise mapping of biomarker–pathology relationships. Spatial concordance between plasma p‐tau217–based measures and regional tau PET signals aligned with Braak stages, supporting biological validity.

In summary, the p‐tau217/Aβ42 ratio emerged as the most informative plasma marker, offering a scalable, biologically grounded tool for AD research and future clinical application.

## CONFLICT OF INTEREST STATEMENT

Authors Yeong Sim Choe, Regina E.Y. Kim, and Donghyeon Kim are employed by the company Neurophet Inc. Hyun Kook Lim is the chief medical officer of Neurophet Inc. Authors Paul Wynveen, Mark Holland, Kinal Bhatt, Zivjena Vucetic, Brian Engel, Corey Carlson, and Andrew Becker are employees of Beckman Coulter Inc., the company that developed and provided the immunoassay platform used in this study. The remaining authors declare that the research was conducted in the absence of any commercial or financial relationships that could be construed as potential conflicts of interest. Any author disclosures are available in the supporting information.

## CONSENT STATEMENT

The study was conducted in accordance with the ethical and safety standards of the local institutional review board of the Catholic University of Korea and the Declaration of Helsinki. All participants provided written informed consent.

## Supporting information



Supporting Information

Supporting Information

## Data Availability

The datasets generated or analyzed during the current study are not publicly available due to the patient data management protocol of Yeouido St. Mary's Hospital but may be obtained from the corresponding author upon reasonable request.

## References

[1] Warmenhoven N , Salvadó G , Janelidze S , et al. A comprehensive head‐to‐head comparison of key plasma phosphorylated Tau 217 biomarker tests. medRxiv. 2024. 10.1093/brain/awae346 PMC11788211 39468767

[2] Feizpour A , Doecke JD , Doré V , et al. Detection and staging of Alzheimer's disease by plasma pTau217 on a high throughput immunoassay platform. eBioMedicine. 2024;109:105405. 39437657 10.1016/j.ebiom.2024.105405 PMC11536028

[3] Vrillon A , Mejía‐Perez JA , Spina S , et al. Association of 18F‐flortaucipir PET with tau neuropathology in AD and other neurodegenerative disorders. Alzheimer Dementia. 2024;20(S2):e092150. 10.1212/WNL.0000000000214576 PMC13085787 41628396

[4] Therriault J , Ashton NJ , Pola I , et al. Comparison of two plasma pTau217 assays to detect and monitor Alzheimer's pathology. eBioMedicine. 2024;102:105046. 38471397 10.1016/j.ebiom.2024.105046 PMC10943661

[5] Palmqvist S , Janelidze S , Quiroz YT , et al. Discriminative Accuracy of plasma phospho‐tau217 for Alzheimer disease vs other neurodegenerative disorders. JAMA. 2020;324(8):772‐781. 32722745 10.1001/jama.2020.12134 PMC7388060

[6] Lehmann S , Gabelle A , Duchiron M , et al. Comparative performance of plasma pTau181/Aβ42, pTau217/Aβ42 ratios, and individual measurements in detecting brain amyloidosis. EBioMedicine. 2025;117:105805. 40513421 10.1016/j.ebiom.2025.105805 PMC12192541

[7] Winslow J , Chenna A , Lo M , et al. Evaluation of plasma pTau217 plus Abeta42/40, and pTau217/Abeta42 ratio as confirmatory tests for amyloid pathology in Alzheimer's disease (AD) (N4.003). Neurology. 2025;104(7_Supplement_1):4073.

[8] Bandara EMS , Asih PR , Pedrini S , Hone E , Fernando WMADB , Martins RN . The role of glial fibrillary acidic protein in the neuropathology of Alzheimer's Disease and Its potential as a blood biomarker for early diagnosis and progression. Mol Neurobiol. 2025;62(12):15576‐15608. 40690136 10.1007/s12035-025-05219-3 PMC12559115

[9] Vasileva‐Metodiev SZ , Spargo D , Klein EG , et al. Diagnostic journey and management of patients with mild cognitive impairment and Alzheimer's disease dementia: a multinational, real‐world survey. J Alzheimers Dis. 2025;104(4):1212‐1234. 40112330 10.1177/13872877251322978 PMC12231793

[10] Rudolph MD , Sutphen CL , Register TC , et al. Associations among plasma, MRI, and amyloid PET biomarkers of Alzheimer's disease and related dementias and the impact of health‐related comorbidities in a community‐dwelling cohort. Alzheimers Dement. 2024;20(6):4159‐4173. 38747525 10.1002/alz.13835 PMC11180870

[11] Dresse MT , Ferreira PCL , Bellaver B , et al. Associations between plasma biomarkers and brain amyloid and tau pathologies in a population‐based cohort without evidence of cognitive impairment. Alzheimers Dement. 2024;20(S2):e092544.

[12] Palmqvist S , Warmenhoven N , Anastasi F , et al. Plasma phospho‐tau217 for Alzheimer's disease diagnosis in primary and secondary care using a fully automated platform. Nat Med. 2025;31(6):2036‐2043. 40205199 10.1038/s41591-025-03622-w PMC12176611

[13] Wang J , Huang S , Lan G , et al. Diagnostic accuracy of plasma pTau217/Aβ42 for Alzheimer's disease in clinical and community cohorts. Alzheimers Dement. 2025;21(3):e70038. 40156286 10.1002/alz.70038 PMC11953589

[14] Jack CR Jr , Andrews JS , Beach TG , Buracchio T , et al. Revised criteria for diagnosis and staging of Alzheimer's disease: alzheimer's Association Workgroup. Alzheimers Dement. 2024;20(8):5143‐5169. doi:10.1002/alz.13859 38934362 PMC11350039

[15] Jennings S . FDA grants breakthrough device designation for Alzheimer's disease. Patient Care (Online). 2025. Accessed August 31, 2025. doi:https://www.patientcareonline.com/view/fda-grants-breakthrough-device-designation-for-alzheimer-s-disease

[16] Li X , Morgan PS , Ashburner J , Smith J , Rorden C . The first step for neuroimaging data analysis: dICOM to NIfTI conversion. J Neurosci Methods. 2016;264:47‐56. 26945974 10.1016/j.jneumeth.2016.03.001

[17] Lee J , Ha S , Kim RE , Lee M , Kim D , Lim HK . Development of amyloid PET analysis pipeline using deep learning‐based brain MRI segmentation—a comparative validation study. Diagnostics. 2022;12(3):623. 35328176 10.3390/diagnostics12030623 PMC8947654

[18] Li Y , Ng YL , Paranjpe MD , et al. Tracer‐specific reference tissues selection improves detection of 18F‐FDG, 18F‐florbetapir, and 18F‐flortaucipir PET SUVR changes in Alzheimer's disease. Hum Brain Mapp. 2022;43(7):2121‐2133. 35165964 10.1002/hbm.25774 PMC8996354

[19] Shang C , Sakurai K , Nihashi T , et al. Comparison of consistency in centiloid scale among different analytical methods in amyloid PET: the CapAIBL, VIZCalc, and Amyquant methods. Ann Nucl Med. 2024;38(6):460‐467. 38512444 10.1007/s12149-024-01919-3 PMC11108942

[20] Dore V , Bullich S , Bohorquez SS , et al. CenTauRz: a standardized quantification of tau PET scans. Alzheimers Dement. 2023;19(S3):e061171.

[21] Lee JH , Lee KU , Lee DY , et al. Development of the Korean version of the consortium to establish a registry for Alzheimer's disease assessment packet (CERAD‐K) clinical and neuropsychological assessment batteries. J Gerontol B Psychol Sci Soc Sci. 2002;57(1):P47‐P53. 11773223 10.1093/geronb/57.1.p47

[22] JH P . Standardization of Korean version of the Mini‐Mental State Examination (MMSE‐K) for use in the elderly. Part II. diagnostic validity. Korean J Neuropsych Assoc. 1989;28:125‐135.

[23] Seo EH , Lee DY , Lee JH , et al. Total scores of the CERAD neuropsychological assessment battery: validation for mild cognitive impairment and dementia patients with diverse etiologies. Am J Geriatr Psychiatry. 2010;18(9):801‐809. 20220577 10.1097/JGP.0b013e3181cab764

[24] Schindler SE , Petersen KK , Saef B , et al. Head‐to‐head comparison of leading blood tests for Alzheimer's disease pathology. Alzheimers Dement. 2024;20(11):8074‐8096. 39394841 10.1002/alz.14315 PMC11567821

[25] Hansson O , Edelmayer RM , Boxer AL , et al. The Alzheimer's Association appropriate use recommendations for blood biomarkers in Alzheimer's disease. Alzheimers Dement. 2022;18(12):2669‐2686. 35908251 10.1002/alz.12756 PMC10087669

[26] Schindler SE , Galasko D , Pereira AC , et al. Acceptable performance of blood biomarker tests of amyloid pathology — recommendations from the Global CEO Initiative on Alzheimer's Disease. Nat Rev Neurol. 2024;20(7):426‐439. 38866966 10.1038/s41582-024-00977-5

[27] Martínez‐Dubarbie F , Guerra‐Ruiz A , López‐García S , et al. Longitudinal trajectory of plasma pTau217 in cognitively unimpaired subjects. Alzheimers Res Ther. 2024;16(1):268. 39702464 10.1186/s13195-024-01642-1 PMC11661039

[28] Snellman A , Ekblad LL , Ashton NJ , et al. Head‐to‐head comparison of plasma pTau181, pTau231 and glial fibrillary acidic protein in clinically unimpaired elderly with three levels of APOE4‐related risk for Alzheimer's disease. Neurobiol Dis. 2023;183:106175. 37268240 10.1016/j.nbd.2023.106175

[29] Meyer MR , Kirmess KM , Eastwood S , Wente‐Roth TL , Irvin F , Holubasch MS , et al. Clinical validation of the PrecivityAD2 blood test: a mass spectrometry‐based test with algorithm combining %p‐tau217 and Aβ42/40 ratio to identify presence of brain amyloid. Alzheimers Dement. 2024;20(5):3179‐3192. doi:10.1002/alz.13764 38491912 PMC11095426

[30] Lehmann S , Schraen‐Maschke S , Vidal J‐S , et al. Clinical value of plasma ALZpath pTau217 immunoassay for assessing mild cognitive impairment. J Neurol Neurosurg Psychiatry. 2024;95(11):1046. 38658136 10.1136/jnnp-2024-333467 PMC11503049

[31] Mattsson‐Carlgren N , Collij LE , Stomrud E , et al. Plasma biomarker strategy for selecting patients with Alzheimer disease for antiamyloid immunotherapies. JAMA Neurol. 2024;81(1):69‐78. 38048096 10.1001/jamaneurol.2023.4596 PMC10696515

[32] Mendes AJ , Ribaldi F , Lathuiliere A , et al. Superiority of pTau217 among plasma biomarkers in diagnostic accuracy in non‐demented persons from the Geneva Memory Center cohort. Alzheimers Dement. 2024;20(S2):e088800.

[33] Insel PS , Mormino EC , Aisen PS , Thompson WK , Donohue MC . Neuroanatomical spread of amyloid β and tau in Alzheimer's disease: implications for primary prevention. Brain Commun. 2020;2(1):fcaa007. 32140682 10.1093/braincomms/fcaa007 PMC7048875

[34] Macedo AC , Tissot C , Therriault J , et al. The use of tau PET to stage alzheimer disease according to the braak staging framework. J Nucl Med. 2023;64(8):1171. 37321820 10.2967/jnumed.122.265200 PMC10394315

[35] Feizpour A , Doré V , Krishnadas N , et al. Alzheimer's disease biological PET staging using plasma p217+tau. Commun Med. 2025;5(1):53. 40016526 10.1038/s43856-025-00768-z PMC11868538

[36] Woo MS , Therriault J , Jonaitis EM , et al. Identification of late‐stage tau accumulation using plasma phospho‐tau217. eBioMedicine. 2024;109:105413. 39500009 10.1016/j.ebiom.2024.105413 PMC11570195

[37] Pereira JB , Janelidze S , Smith R , et al. Plasma GFAP is an early marker of amyloid‐β but not tau pathology in Alzheimer's disease. Brain. 2021;144(11):3505‐3516. 34259835 10.1093/brain/awab223 PMC8677538

[38] Youn W , Yun M , Lee CJ , Schöll M . Cautions on utilizing plasma GFAP level as a biomarker for reactive astrocytes in neurodegenerative diseases. Mol Neurodegener. 2025;20(1):54. 40346659 10.1186/s13024-025-00846-9 PMC12065376

[39] Talwar P , Kushwaha S , Chaturvedi M , Mahajan V . Systematic review of different neuroimaging correlates in mild cognitive impairment and Alzheimer's disease. Clin Neuroradiol. 2021;31(4):953‐967. 34297137 10.1007/s00062-021-01057-7

[40] Mitolo M , Lombardi G , Manca R , Nacmias B , Venneri A . Association between blood‐based protein biomarkers and brain MRI in the Alzheimer's disease continuum: a systematic review. J Neurol. 2024;271(11):7120‐7140. 39264441 10.1007/s00415-024-12674-w PMC11560990

[41] Sánchez‐Juan P , Valeriano‐Lorenzo E , Ruiz‐González A , et al. Serum GFAP levels correlate with astrocyte reactivity, post‐mortem brain atrophy and neurofibrillary tangles. Brain. 2024;147(5):1667‐1679. 38634687 10.1093/brain/awae035 PMC11068326

[42] Bellaver B , Povala G , Ferreira PCL , et al. Plasma GFAP for populational enrichment of clinical trials in preclinical Alzheimer's disease. Alzheimers Dement. 2025;21(5):e70209. 40346617 10.1002/alz.70209 PMC12064411

[43] Babulal GM . Inclusion of ethnoracial populations and diversity remains a key challenge in Alzheimer's disease biofluid‐based biomarker studies. J Neurol Sci. 2021;421:117269. 33357998 10.1016/j.jns.2020.117269 PMC7914213

