# Supplementary material for: Neuroimaging correlates and biomarker performance of a fully automated plasma p‐tau217/Aβ42 ratio assay in a clinical cohort with Alzheimer's disease
Source: Alzheimers Dement. 2025 Nov 24;21(11):e70942. doi: 10.1002/alz.70942 (PMC12644925; doi:10.1002/alz.70942)
Supplement: Supplementary file 2 — Supporting Information [file ALZ-21-e70942-s002.docx]

**Supplementary Materials for “Neuroimaging correlates and biomarker performance of a fully automated plasma pTau217/Aβ42 ratio assay in a clinical cohort with Alzheimer’s disease"” by Um et al.**

**Supplementary Methods**

Supplementary Method S1. Diagnostic classification and exclusion criteria

Supplementary Method S2. Apolipoprotein E genotyping

Supplementary Method S3. Tau PET region mapping to Braak Stage

Supplementary Method S4. Statistical software details

**Supplementary Tables**

Supplementary Table S1. Adjusted plasma biomarker levels (mean ± SD) across diagnostic groups (ANCOVA adjusted for age & sex)

Supplementary Table S2. Comparison of plasma biomarker levels between APOE4 carriers and non-carriers

Supplementary Table S3. Comparative performance of plasma biomarkers with and without APOE4 inclusion in all subjects(N=238)

Supplementary Table S4. Comparative performance of plasma biomarkers with and without APOE4 inclusion in cognitively unimpaired group (Aβ-CU and Aβ+CU) (N=94)

Supplementary Table S5. Comparative performance of plasma biomarkers with and without APOE4 inclusion in cognitively impaired group (MCI and AD dementia) (N=144)

Supplementary Table S6. Significant clusters showing negative associations between Aβ42 levels and PET signal

Supplementary Table S7. Significant clusters showing associations between pTau217 levels, PET signals and structural MRI measures

Supplementary Table S8. Significant clusters showing associations between plasma pTau217/ Aβ42 levels, PET signals and structural MRI measures

Supplementary Table S9. Significant clusters showing associations between plasma GFAP levels, PET signals and structural MRI measures

Supplementary Table S10. Partial correlation coefficients between plasma biomarkers and regional tau PET SUVRs based on Braak stage(N=76)

Supplementary Table S11. Partial correlation coefficients between plasma biomarkers and regional tau PET SUVRs in the cognitively unimpaired (Aβ-CU and Aβ+CU) group (N=24)

Supplementary Table S12. Partial correlation coefficients between plasma biomarkers and regional tau PET SUVRs in the in the cognitively impaired (MCI+AD dementia) group (N=52)

**Supplementary Figures**

Supplementary Figure S1. ROC curve comparison for a) biomarker-only vs. b) biomarker+APOE4 models in NC and preclinical AD group (N=94)

Supplementary Figure S2. ROC curve comparison for a) biomarker-only vs. b) biomarker+APOE4 models in MCI and AD dementia group(N=144)

Supplementary Figure S3. Partial correlations between plasma biomarkers and tau PET SUVRs in the cognitively unimpaired (Aβ-CU and Aβ+CU) group (N=24)

Supplementary Figure S4. Partial correlations between plasma biomarkers and tau PET SUVRs in the cognitively impaired (MCI+AD dementia) group (N=52)

**Supplementary References**

**Supplementary Methods**

**Supplementary Method S1. Diagnostic classification and exclusion criteria**

This investigation encompassed a cohort of 262 elderly individuals recruited from the Catholic Aging Brain Imaging Database (CABID). The participants were patients who attended the Catholic Brain Health Center, Yeouido St. Mary’s Hospital, The Catholic University of Korea, during the period spanning from 2022-2024. Eligible participants were those aged 55 years or older who underwent magnetic resonance imaging (MRI), positron emission tomography (PET), and comprehensive clinical evaluations, blood sampling for plasma biomarkers, which included the Korean Version of the Consortium to Establish a Registry for Alzheimer’s Disease Assessment Packet (CERAD-K)^1,^ and blood sampling for plasma biomarkers. Neuroimaging and blood sampling were conducted within a maximum interval of two years.

Participants were categorized into four diagnostic groups predicated upon clinical evaluations and biomarker evidence, adhering to the recent revised diagnostic and staging criteria from Alzheimer’s Association workgroup.^2^

1. Amyloid-beta (Aβ) negative and cognitively unimpaired (Aβ−CU): Participants exhibiting a clinical dementia rating (CDR)^3^ score of 0, devoid of clinical indications of cognitive impairment, and demonstrating negative amyloid PET results suggestive of the absence of Aβ accumulation.

2. Aβ positive and cognitively unimpaired (Aβ+ CU) Participants with a CDR score of 0, lacking objective cognitive deficits on neuropsychological assessment, yet displaying positive amyloid PET results indicative of biomarker evidence for Aβ deposition.

3. Aβ negative mild cognitive impairment (Aβ -MCI): Defined according to Petersen’s criteria^4^, participants exhibited objective cognitive deficits on neuropsychological testing, with performance in at least one of the eight CERAD-K domains more than 1.5 standard deviations below age- and education-adjusted norms. They also had preserved general cognitive function, intact activities of daily living with a CDR score of 0.5, and negative amyloid PET findings indicative of Aβ deposition.

4. Aβ positive mild cognitive impairment (Aβ+-MCI): Defined according to Petersen’s criteria^4^, participants exhibited objective cognitive deficits on neuropsychological testing, with performance in at least one of the eight CERAD-K domains more than 1.5 standard deviations below age- and education-adjusted norms. They also had preserved general cognitive function, intact activities of daily living with a CDR score of 0.5, and positive amyloid PET findings indicative of Aβ deposition.

5. Alzheimer’s disease dementia (AD dementia): Diagnosis was rendered based on established clinical guidelines, with a CDR score of 1 or greater, indicating that participants experienced substantial cognitive and functional decline consistent with dementia, accompanied by amyloid PET evidence of Aβ deposition.

Participants were excluded from the study if they satisfied any of the following criteria. Individuals with neurological disorders, encompassing non-AD neurodegenerative conditions such as Parkinson’s disease, Lewy body dementia, or frontotemporal dementia, were deemed ineligible. Furthermore, those with a history of cerebrovascular accident, epilepsy, normal pressure hydrocephalus, or significant traumatic brain injury were excluded from participation. Participants presenting with major psychiatric disorders were also ineligible, including those diagnosed with active major depressive disorder, bipolar disorder, schizophrenia, or other psychotic disorders as classified by the Diagnostic and Statistical Manual of Mental Disorders, Fifth Edition. Additionally, individuals with severe anxiety disorders or a history of substance use disorders within the preceding year were excluded. The exclusion criteria further encompassed medical conditions that could potentially impair cognitive function, such as uncontrolled diabetes, thyroid dysfunction, renal insufficiency, hepatic dysfunction, or chronic inflammatory diseases. Individuals with active malignancies, with the exception of treated basal cell carcinoma, were also excluded. Lastly, participants possessing contraindications to neuroimaging were not eligible for inclusion. This included individuals unable to undergo magnetic resonance imaging due to the presence of metal implants or severe claustrophobia, as well as those with contraindications to positron emission tomography imaging, such as an allergy to radiotracers.

**Supplementary Method S2. Apolipoprotein E genotyping**

In order to ascertain the apolipoprotein E genotype genotype, deoxyribonucleic acid (DNA) was isolated from the blood samples of participants utilizing the QIAamp Blood DNA Maxi Kit (Qiagen, Valencia, CA, USA). TaqMan single nucleotide polymorphism (SNP) genotyping assays (Applied Biosystems, Foster City, CA, USA) were employed to detect two significant APOE SNPs: rs429358 (ε 4) and rs7412 (ε 2). Participants were categorized as carriers of the apolipoprotein E ε4 allele(APOE4) if they possessed a minimum of one ε4 allele, while those who lacked the ε4 allele were classified as non-carriers of APOE4.

**Supplementary Method S3. Tau PET region mapping to Braak Stage**

| Braak Stage | Region | Definition |
| --- | --- | --- |
| I-II | Mesial temporal (CenTaur) | Early tau deposition; distinguishes normal aging vs. early AD |
|  | Left mesial temporal SUVR |  |
|  | Right mesial temporal SUVR |  |
| III-IV | Meta-temporal (CenTaur) | Tau propagation in MCI; mid-stage AD pathology |
|  | Left meta-temporal SUVR |  |
|  | Right meta-temporal SUVR |  |
|  | Frontal cortex (CenTaur) | Executive dysfunction; limbic-neocortical spread |
|  | Left frontal SUVR |  |
|  | Right frontal SUVR |  |
| V-VI | Temporo-parietal (CenTaur) | Temporo-parietal association cortex  Widespread cortical tau deposition; late-stage AD |
|  | Left temporo-parietal SUVR |  |
|  | Right temporo-parietal SUVR |  |
| Overall Tau burden | Global neocortex (CenTaur) | Overall Tau burden |
|  | Left hemisphere SUVR |  |
|  | Right hemisphere SUVR |  |

**Supplementary Method S4. Statistical software details**

All statistical analyses were primarily implemented utilizing Python (version 3.9). Descriptive statistics, group comparisons (t-test, analysis of variance, and analysis of covariance), and Bonferroni-corrected post-hoc comparisons were conducted using the ‘statsmodel’ and ‘scipy’ packages. To validate and reproduce diagnostic performance metrics, independent analyses were also conducted using R (version 4.1.3). The ‘cutpointr’ package was utilized to generate receiver operative characteristics curves and threshold optimization, while the ‘epiR’ package was adopted to calculate binary calssficiation metrics including sensitivity, specificity, positive and negative predictive values and likelihood ratios. Logistic regression models were also fit using the ‘stats’ package in R. Surface-based vertex-wise analyses were performed using FreeSurfer (version 7.2.0), including general linear models (GLM) applied to cortical positron emission tomography(PET) and structural MRI data. A smoothing kernel of 5 mm full width at half maximum (FWHM) was applied for PET data and 10 mm for MRI data. Correction for multiple comparisons was performed using Monte Carlo simulation (10,000 iterations) via the `mri_glmfit-sim` tool in FreeSurfer. Neuroimaging data were preprocessed and visualized using FreeSurfer and Python packages including `nibabel` and `nilearn`. Partial correlation analyses were performed to assess the association between plasma biomarkers and Braak stage-related tau PET standardized uptake value ratios, adjusting for age and sex using a residual-based method; results were visualized as heatmaps with ‘seaborn’ and ‘matplotlib’.

**Supplementary Tables**

**Supplementary Table S1. Adjusted plasma biomarker levels (mean ± SD) across diagnostic groups**

|  | Aβ−CU  (N=77) | Aβ+ CU  (N=24) | Aβ− MCI  (N=62) | Aβ+ MCI  (N=78) | AD dementia  (N=21) | p-value |
| --- | --- | --- | --- | --- | --- | --- |
| pTau217(pg/mL) | 0.550 ± 0.355 | 1.147 ± 0.458 | 0.795 ± 0.605 | 1.746 ± 1.688 | 1.952 ± 1.105 | <0.001 |
| Aβ42(pg/mL) | 27.636 ± 6.909 | 23.542 ± 4.634 | 28.774 ± 6.810 | 24.359 ± 6.457 | 22.619 ± 5.563 | <0.001 |
| pTau217/ Aβ42 ratio | 0.020 ± 0.013 | 0.050 ± 0.021 | 0.029 ± 0.022 | 0.069 ± 0.038 | 0.086 ± 0.034 | <0.001 |
| GFAP (pg/mL) | 12.299 ± 7.356 | 20.667 ± 10.627 | 16.516 ± 8.413 | 21.551 ± 9.533 | 22.810 ± 11.044 | <0.001 |

Values represent estimated marginal means from ANCOVA models adjusted for age and sex.

***Abbreviations:*** SD=standard deviation; ANCOVA=Analysis of covariance, Aβ=amyloid beta; CU=cognitively unimpaired; AD= Alzheimer’s disease; MCI = mild cognitive impairment; pTau217 = plasma phosphorylated tau at threonine 217; Aβ42 = plasma amyloid-beta 42; GFAP = plasma glial fibrillary acidic protein

**Supplementary Table S2. Comparison of plasma biomarker levels between APOE4 carriers and non-carriers**

|  | APOE4 Non-carrier  (N=160) | APOE4 Carrier  (n=102) | p-value |
| --- | --- | --- | --- |
| pTau217(pg/mL) | 0.943 ± 0.755 | 1.425 ± 1.580 | 0.005 |
| Aβ42(pg/mL) | 27.606 ± 6.712 | 23.873 ± 6.310 | <0.001 |
| pTau217/ Aβ42 ratio | 0.036 ± 0.028 | 0.059 ± 0.042 | <0.001 |
| GFAP (pg/mL) | 16.850 ± 9.772 | 18.931 ± 9.575 | 0.090 |

Plasma levels of amyloid-beta 42 (Aβ42), phosphorylated tau at threonine 217 (pTau217), amyloid-beta 42 (Aβ42), pTau217/ Aβ42 ratio and glial fibrillary acidic protein (GFAP) were compared between apolipoprotein E ε4 allele (APOE4) carriers and non-carriers independent t-test. Data are presented as mean ± standard deviation (SD).

**Supplementary Table S3. Comparative performance of plasma biomarkers with and without APOE4 inclusion in all subjects(N=238)**

| Biomarker | AUC (Biomarker Only) | 95% CI (Biomarker Only) | AUC (+APOE4) | 95% CI (+APOE4) |
| --- | --- | --- | --- | --- |
| pTau217 | 0.913 | 0.868–0.953 | 0.914 | 0.867–0.952 |
| Aβ42 | 0.748 | 0.680–0.814 | 0.758 | 0.692-0.816 |
| pTau217/Aβ42 ratio | 0.943 | 0.903-0.973 | 0.944 | 0.906-0.974 |
| GFAP | 0.813 | 0.754–0.864 | 0.842 | 0.787–0.889 |

All AUCs were derived from logistic regression models classifying amyloid PET positivity. The 95% CI was estimated using 1000 bootstrap iterations. ROC curve analysis was performed in all subjects with Centiloid (CL) > 20 as the amyloid PET positivity threshold.

*Abbreviations*: ROC=receiver operating characteristics; NC=normal cognition; AD=Alzheimer’s disease; pTau217 = phosphorylated tau at threonine 217; Aβ42 =amyloid-beta 42; GFAP =glial fibrillary acidic protein; AUC = area under the curve; CI = confidence interval; APOE4 = apolipoprotein E ε4 allele

**Supplementary Table S4. Comparative performance of plasma biomarkers with and without APOE4 inclusion in cognitively unimpaired group (Aβ−CU and Aβ+CU) (N=94)**

| Biomarker | AUC (Biomarker Only) | 95% CI (Biomarker Only) | AUC (+APOE4) | 95% CI (+APOE4) |
| --- | --- | --- | --- | --- |
| pTau217 | 0.907 | 0.838-0.964 | 0.907 | 0.840-0.962 |
| Aβ42 | 0.718 | 0.610-0.823 | 0.720 | 0.610-0.822 |
| pTau217/Aβ42 ratio | 0.945 | 0.899-0.983 | 0.944 | 0.899-0.982 |
| GFAP | 0.836 | 0.740-0.924 | 0.851 | 0.748-0.933 |

All AUCs were derived from logistic regression models classifying amyloid PET positivity. The 95% CI was estimated using 1000 bootstrap iterations. ROC curve analysis was performed in NC and preclinical AD with Centiloid (CL) > 20 as the amyloid PET positivity threshold.

*Abbreviations*: ROC=receiver operating characteristics; Aβ=amyloid beta; CU=cognitively unimpaired; pTau217 = phosphorylated tau at threonine 217; Aβ42 = amyloid-beta 42; GFAP = glial fibrillary acidic protein; AUC = area under the curve; CI = confidence interval; APOE4 = apolipoprotein E ε4 allele

**Supplementary Table S5. Comparative performance of plasma biomarkers with and without APOE4 inclusion in cognitively impaired group (MCI and AD dementia) (N=144)**

| Biomarker | AUC (Biomarker Only) | 95% CI (Biomarker Only) | AUC (+APOE4) | 95% CI (+APOE4) |
| --- | --- | --- | --- | --- |
| pTau217 | 0.879 | 0.783-0.963 | 0.874 | 0.786–0.957 |
| Aβ42 | 0.785 | 0.693–0.870 | 0.803 | 0.712–0.887 |
| pTau217/Aβ42 ratio | 0.907 | 0.820–0.975 | 0.906 | 0.818–0.974 |
| GFAP | 0.737 | 0.631–0.835 | 0.804 | 0.700–0.893 |

All AUCs were derived from logistic regression models classifying amyloid PET positivity. The 95% CI was estimated using 1000 bootstrap iterations. ROC curve analysis was performed in MCI and AD dementia with Centiloid (CL) > 20 as the amyloid PET positivity threshold.

*Abbreviations*: ROC=receiver operating characteristics; MCI=mild cognitive impairment; AD=Alzheimer’s disease; pTau217 = phosphorylated tau at threonine 217; Aβ42 = amyloid-beta 42; GFAP = glial fibrillary acidic protein; AUC = area under the curve; CI = confidence interval; APOE4 = apolipoprotein E ε4 allele

**Supplementary Table S6. Significant clusters showing negative associations between Aβ42 levels and PET signal**

| Hemisphere | Measure | FWHM | Threshold | Region | Max t-value | Cluster Size (mm²) | MNI-X | MNI-Y | MNI-Z | CWP |
| --- | --- | --- | --- | --- | --- | --- | --- | --- | --- | --- |
| Left | Amyloid PET  PET | 5 | 1.3 | Rostral middle frontal | –6.564 | 63,466.16 | –37.4 | 50.7 | –2.2 | <0.001 |
| Right | Amyloid PET  PET | 5 | 1.3 | Medial orbitofrontal | –7.661 | 64,428.73 | 9.4 | 45.2 | –8.9 | <0.001 |

Clusters showing significant negative associations between Aβ42 levels and cortical amyloid PET signal. Analyses were performed using a surface-based general linear model with a 5 mm FWHM smoothing kernel and a vertex-wise threshold of |t| > 1.3. Correction for multiple comparisons was applied using a cluster-wise method based on Monte Carlo simulation, as implemented in FreeSurfer. Reported coordinates correspond to the peak vertex of each cluster in MNI space.

*Abbreviations:* Aβ42= amyloid-beta 42; PET=positron emission tomography; FWHM=full width at half maximum; MNI=Montreal Neurological Institute; CWP= cluster-wise p-value.

**Supplementary Table S7. Significant clusters showing associations between pTau217 levels, PET signals and structural MRI measures**

| Hemisphere | Measure | FWHM | Threshold | Region | Max t-value | Cluster Size (mm²) | MNI-X | MNI-Y | MNI-Z | CWP |
| --- | --- | --- | --- | --- | --- | --- | --- | --- | --- | --- |
| Left | Amyloid PET | 5 | 1.3 | Precuneus | 17.692 | 76183.59 | -14.3 | -41.1 | 42.7 | <0.001 |
| Right | Amyloid PET | 5 | 1.3 | Supramarginal | 23.13 | 75645.87 | 52.4 | -45.1 | 20.2 | <0.001 |
| Right | Tau PET | 5 | 1.3 | Inferior parietal | 4.079 | 1987.33 | 44.8 | -47.1 | 18.1 | 0.004 |
| Left | Volume | 10 | 1.3 | Parahippocampal | -4.91 | 6337.73 | -32 | -40.9 | -9.5 | <0.001 |
| Left | Volume | 10 | 1.3 | Superior frontal | -3.054 | 2843.53 | -18.8 | 32.4 | 49.8 | <0.001 |
| Left | Volume | 10 | 1.3 | Posterior cingulate | -3.307 | 2166.69 | -17.8 | -30.7 | 38.1 | <0.001 |
| Left | Volume | 10 | 1.3 | Supramarginal | -2.697 | 1314.08 | -59.3 | -34.4 | 37.4 | 0.004 |
| Left | Volume | 10 | 1.3 | Caudal middle frontal | -4.607 | 1217.65 | -34.9 | 25.2 | 42.6 | 0.008 |
| Left | Volume | 10 | 1.3 | Inferior parietal | -4.239 | 1082.68 | -47.7 | -60 | 41.6 | 0.016 |
| Right | Volume | 10 | 1.3 | Fusiform | -6.409 | 10396.88 | 37.7 | -28.6 | -20.4 | <0.001 |
| Right | Volume | 10 | 1.3 | Medial orbitofrontal | -4.584 | 5680.3 | 8.5 | 59 | -1.6 | <0.001 |
| Right | Volume | 10 | 1.3 | Precuneus | -3.606 | 1410.95 | 9.1 | -67.8 | 35.6 | 0.002 |
| Left | Thickness | 10 | 1.3 | Precentral | -4.438 | 10456.88 | -41.8 | 1.8 | 23.9 | <0.001 |
| Left | Thickness | 10 | 1.3 | Parahippocampal | -3.885 | 6430.34 | -25.2 | -31.2 | -10.4 | <0.001 |
| Left | Thickness | 10 | 1.3 | Superior frontal | -3.351 | 4125.29 | -14.1 | 32.2 | 51.1 | <0.001 |
| Left | Thickness | 10 | 1.3 | Inferior temporal | -4.242 | 4072.3 | -56.5 | -41.2 | -13.1 | <0.001 |
| Left | Thickness | 10 | 1.3 | Entorhinal | -2.569 | 1473.79 | -21.9 | -21.1 | -26.8 | 0.010 |
| Left | Thickness | 10 | 1.3 | Posterior cingulate | -2.96 | 1221.19 | -7.5 | -24.4 | 36.7 | 0.016 |
| Right | Thickness | 10 | 1.3 | Lateral orbitofrontal | -3.807 | 4231.83 | 24.5 | 35.9 | -14.4 | <0.001 |
| Right | Thickness | 10 | 1.3 | Pars triangularis | -3.509 | 1910.15 | 55.7 | 21.1 | 18.4 | 0.002 |
| Right | Thickness | 10 | 1.3 | Parahippocampal | -2.738 | 1113.11 | 24 | -29.1 | -11.8 | 0.008 |

Clusters showing significant associations between pTau217 levels, PET signals (amyloid PET and tau PET), and cortical measures (cortical volume and thickness). Analyses were performed using a surface-based general linear model with a 5 mm or 10 mm FWHM smoothing kernel depending on the modality and a vertex-wise threshold of |t| > 1.3. Correction for multiple comparisons was applied using a cluster-wise method based on Monte Carlo simulation, as implemented in FreeSurfer. Reported coordinates correspond to the peak vertex of each cluster in MNI space.

*Abbreviations:* pTau217= phosphorylated tau at threonine 217; PET= positron emission tomography; MRI=magnetic resonance imaging; FWHM=full width at half maximum; MNI=Montreal Neurological Institute; CWP=cluster-wise p-value.

**Supplementary Table S8. Significant clusters showing associations between plasma pTau217/ Aβ42 levels, PET signals and structural MRI measures**

| Hemisphere | Measure | FWHM | Threshold | Region | Max t-value | Cluster Size (mm²) | MNI-X | MNI-Y | MNI-Z | CWP |
| --- | --- | --- | --- | --- | --- | --- | --- | --- | --- | --- |

| Left | Amyloid PET | 5 | 1.3 | Precuneus | 32.829 | 76148.65 | -9.0 | -40.3 | 39.1 | <0.001 |
| --- | --- | --- | --- | --- | --- | --- | --- | --- | --- | --- |
| Right | Amyloid PET | 5 | 1.3 | Precuneus | 34.144 | 75665.60 | 14.4 | -51.9 | 36.1 | <0.001 |
| Left | Tau PET | 5 | 1.3 | Middle temporal | 6.695 | 60295.88 | -48.1 | -38.0 | -5.7 | <0.001 |
| Right | Tau PET | 5 | 1.3 | Inferior parietal | 9.157 | 59976.02 | 44.9 | -46.6 | 17.6 | <0.001 |
| Left | Volume | 10 | 1.3 | Entorhinal | -7.096 | 16865.02 | -19.8 | -14.7 | -28.1 | <0.001 |
| Left | Volume | 10 | 1.3 | Medial orbitofrontal | -5.591 | 13397.89 | -11.7 | 41.0 | -3.3 | <0.001 |
| Left | Volume | 10 | 1.3 | Caudal middle frontal | -5.522 | 1987.45 | -34.6 | 25.7 | 40.1 | <0.001 |
| Right | Volume | 10 | 1.3 | Middle temporal | -8.382 | 33448.46 | 63.1 | -38.1 | -12.1 | <0.001 |
| Left | Thickness | 10 | 1.3 | Banks of the superior temporal sulcus | -8.957 | 46515.19 | -42.5 | -51.4 | 11.4 | <0.001 |
| Right | Thickness | 10 | 1.3 | Superior frontal | -10.214 | 50899.17 | 20.1 | 22.8 | 45.3 | <0.001 |

Clusters showing significant associations between pTau217/ Aβ42 levels, PET signals (amyloid PET and tau PET), and cortical measures (cortical volume and thickness). Analyses were performed using a surface-based general linear model with a 5 mm or 10 mm FWHM smoothing kernel depending on the modality and a vertex-wise threshold of |t| > 1.3. Correction for multiple comparisons was applied using a cluster-wise method based on Monte Carlo simulation, as implemented in FreeSurfer. Reported coordinates correspond to the peak vertex of each cluster in MNI space.

*Abbreviations:* pTau217= phosphorylated tau at threonine 217; Aβ42= amyloid-beta 42; PET= positron emission tomography; MRI=magnetic resonance imaging; FWHM=full width at half maximum; MNI=Montreal Neurological Institute; CWP=cluster-wise p-value.

**Supplementary Table S9. Significant clusters showing associations between plasma GFAP levels, PET signals and structural MRI measures**

| **Hemisphere** | **Measure** | **FWHM** | **Threshold** | **Region** | **Max t-value** | **Cluster Size (mm²)** | **MNI-X** | **MNI-Y** | **MNI-Z** | **CWP** |
| --- | --- | --- | --- | --- | --- | --- | --- | --- | --- | --- |
| Left | Amyloid PET | 5 | 1.3 | Precuneus | 11.501 | 72063.38 | -8.9 | -42.9 | 41.2 | <0.001 |
| Right | Amyloid PET | 5 | 1.3 | Precuneus | 11.934 | 70211.28 | 7.4 | -56.7 | 50.1 | <0.001 |
| Left | Tau PET | 5 | 1.3 | Parahippocampal | 5.024 | 50722.42 | -21.4 | -23.8 | -23.6 | <0.001 |
| Right | Tau PET | 5 | 1.3 | Insula | 4.300 | 20999.98 | 36.0 | -18.5 | -2.4 | <0.001 |
| Right | Tau PET | 5 | 1.3 | Entorhinal | 3.893 | 19603.70 | 30.9 | -13.9 | -31.1 | <0.001 |
| Left | Volume | 10 | 1.3 | Fusiform | -8.254 | 13351.67 | -34.0 | -34.7 | -22.0 | <0.001 |
| Left | Volume | 10 | 1.3 | Superior frontal | -4.958 | 6274.14 | -12.3 | 64.0 | 8.8 | <0.001 |
| Left | Volume | 10 | 1.3 | Precuneus | -4.170 | 2869.45 | -7.8 | -58.8 | 24.9 | <0.001 |
| Left | Volume | 10 | 1.3 | Rostral middle frontal | -3.849 | 1856.02 | -31.1 | 27.3 | 39.5 | <0.001 |
| Right | Volume | 10 | 1.3 | Fusiform | -8.243 | 8386.00 | 35.8 | -32.8 | -21.7 | <0.001 |
| Right | Volume | 10 | 1.3 | Medial orbitofrontal | -6.038 | 3649.27 | 8.5 | 59.0 | -1.6 | <0.001 |
| Right | Volume | 10 | 1.3 | Precuneus | -3.949 | 2374.34 | 5.2 | -65.7 | 32.4 | <0.001 |
| Right | Volume | 10 | 1.3 | Caudal middle frontal | -3.926 | 1152.71 | 37.8 | 22.6 | 40.0 | 0.011 |
| Right | Volume | 10 | 1.3 | Supramarginal | -3.814 | 1037.68 | 53.6 | -32.0 | 44.2 | 0.024 |
| Left | Thickness | 10 | 1.3 | Fusiform | -7.964 | 45244.43 | -32.9 | -35.0 | -22.6 | <0.001 |
| Right | Thickness | 10 | 1.3 | Superior frontal | -7.380 | 42911.97 | 19.9 | 23.3 | 46.4 | <0.001 |

Clusters showing significant associations between GFAP levels, PET signals (amyloid PET and tau PET), and cortical measures (cortical volume and thickness). Analyses were performed using a surface-based general linear model with a 5 mm or 10 mm FWHM smoothing kernel depending on the modality and a vertex-wise threshold of |t| > 1.3. Correction for multiple comparisons was applied using a cluster-wise method based on Monte Carlo simulation, as implemented in FreeSurfer. Reported coordinates correspond to the peak vertex of each cluster in MNI space.

*Abbreviations* GFAP = glial fibrillary acidic protein; PET= positron emission tomography; MRI=magnetic resonance imaging; FWHM=full width at half maximum; MNI=Montreal Neurological Institute; CWP=cluster-wise p-value.

**Supplementary Table S10. Partial correlation coefficients between plasma biomarkers and regional tau PET SUVRs based on Braak stage**

| Biomarker | Region | r | p |
| --- | --- | --- | --- |
| pTau217 | Left mesial temporal SUVR | 0.29 | 0.012 |
| pTau217 | Mesial temporal (CenTaur) | 0.31 | 0.007 |
| pTau217 | Right mesial temporal SUVR | 0.31 | 0.006 |
| pTau217 | Frontal cortex (CenTaur) | 0.24 | 0.035 |
| pTau217 | Left frontal SUVR | 0.24 | 0.035 |
| pTau217 | Left meta-temporal SUVR | 0.26 | 0.025 |
| pTau217 | Meta-temporal (CenTaur) | 0.26 | 0.023 |
| pTau217 | Right frontal SUVR | 0.23 | 0.043 |
| pTau217 | Right meta-temporal SUVR | 0.25 | 0.028 |
| pTau217 | Left temporo-parietal SUVR | 0.27 | 0.018 |
| pTau217 | Right temporo-parietal SUVR | 0.26 | 0.023 |
| pTau217 | Temporo-parietal (CenTaur) | 0.27 | 0.018 |
| pTau217 | Global neocortex (CenTaur) | 0.27 | 0.017 |
| pTau217 | Left hemisphere SUVR | 0.27 | 0.027 |
| pTau217 | Right hemisphere SUVR | 0.27 | 0.020 |
| Aβ42 | Left mesial temporal SUVR | -0.20 | 0.077 |
| Aβ42 | Mesial temporal (CenTaur) | -0.20 | 0.084 |
| Aβ42 | Right mesial temporal SUVR | -0.19 | 0.098 |
| Aβ42 | Frontal cortex (CenTaur) | -0.22 | 0.058 |
| Aβ42 | Left frontal SUVR | -0.21 | 0.074 |
| Aβ42 | Left meta-temporal SUVR | -0.21 | 0.065 |
| Aβ42 | Meta-temporal (CenTaur) | -0.21 | 0.063 |
| Aβ42 | Right frontal SUVR | -0.22 | 0.053 |
| Aβ42 | Right meta-temporal SUVR | -0.21 | 0.073 |
| Aβ42 | Left temporo-parietal SUVR | -0.19 | 0.101 |
| Aβ42 | Right temporo-parietal SUVR | -0.20 | 0.079 |
| Aβ42 | Temporo-parietal (CenTaur) | -0.20 | 0.081 |
| Aβ42 | Global neocortex (CenTaur) | -0.22 | 0.058 |
| Aβ42 | Left hemisphere SUVR | -0.21 | 0.070 |
| Aβ42 | Right hemisphere SUVR | -0.22 | 0.056 |
| pTau217/Aβ42 ratio | Left mesial temporal SUVR | 0.55 | <0.001 |
| pTau217/Aβ42 ratio | Mesial temporal (CenTaur) | 0.56 | <0.001 |
| pTau217/Aβ42 ratio | Right mesial temporal SUVR | 0.57 | <0.001 |
| pTau217/Aβ42 ratio | Frontal cortex (CenTaur) | 0.53 | <0.001 |
| pTau217/Aβ42 ratio | Left frontal SUVR | 0.52 | <0.001 |
| pTau217/Aβ42 ratio | Left meta-temporal SUVR | 0.56 | <0.001 |
| pTau217/Aβ42 ratio | Meta-temporal (CenTaur) | 0.57 | <0.001 |
| pTau217/Aβ42 ratio | Right frontal SUVR | 0.52 | <0.001 |
| pTau217/Aβ42 ratio | Right meta-temporal SUVR | 0.55 | <0.001 |
| pTau217/Aβ42 ratio | Left temporo-parietal SUVR | 0.56 | <0.001 |
| pTau217/Aβ42 ratio | Right temporo-parietal SUVR | 0.55 | <0.001 |
| pTau217/Aβ42 ratio | Temporo-parietal (CenTaur) | 0.56 | <0.001 |
| pTau217/Aβ42 ratio | Global neocortex (CenTaur) | 0.58 | <0.001 |
| pTau217/Aβ42 ratio | Left hemisphere SUVR | 0.57 | <0.001 |
| pTau217/Aβ42 ratio | Right hemisphere SUVR | 0.57 | <0.001 |
| GFAP | Left mesial temporal SUVR | 0.45 | <0.001 |
| GFAP | Mesial temporal (CenTaur) | 0.46 | <0.001 |
| GFAP | Right mesial temporal SUVR | 0.46 | <0.001 |
| GFAP | Frontal cortex (CenTaur) | 0.35 | 0.002 |
| GFAP | Left frontal SUVR | 0.35 | 0.002 |
| GFAP | Left meta-temporal SUVR | 0.44 | <0.001 |
| GFAP | Meta-temporal (CenTaur) | 0.43 | <0.001 |
| GFAP | Right frontal SUVR | 0.34 | 0.003 |
| GFAP | Right meta-temporal SUVR | 0.40 | <0.001 |
| GFAP | Left temporo-parietal SUVR | 0.41 | <0.001 |
| GFAP | Right temporo-parietal SUVR | 0.36 | 0.001 |
| GFAP | Temporo-parietal (CenTaur) | 0.39 | <0.001 |
| GFAP | Global neocortex (CenTaur) | 0.41 | <0.001 |
| GFAP | Left hemisphere SUVR | 0.42 | <0.001 |
| GFAP | Right hemisphere SUVR | 0.38 | 0.001 |

This table presents partial correlation coefficients (r) and corresponding p-values between four plasma biomarkers (pTau217, Aβ42, pTau217/Aβ42 ratio, and GFAP) and tau PET SUVRs derived from Braak stage-related cortical regions. All correlations were adjusted for age and sex using linear regression residuals.

*Abbreviations:* pTau217=phosphorylated tau at threonine 217; Aβ42=amyloid-β42; GFAP=glial fibrillary acidic protein; SUVR=standardized uptake value ratio; PET=positron emission tomography.

**Supplementary Table S11. Partial correlation coefficients between plasma biomarkers and regional tau PET SUVRs in the cognitively unimpaired group (Aβ−CU and Aβ+CU) (N=24)**

| Biomarker | Region | r | p |
| --- | --- | --- | --- |
| Aβ42 | Frontal cortex (CenTaur) | -0.4 | 0.054 |
| Aβ42 | Left frontal SUVR | -0.36 | 0.086 |
| Aβ42 | Right frontal SUVR | -0.43 | 0.034 |
| Aβ42 | Mesial temporal (CenTaur) | -0.45 | 0.028 |
| Aβ42 | Left mesial temporal SUVR | -0.48 | 0.018 |
| Aβ42 | Right mesial temporal SUVR | -0.4 | 0.054 |
| Aβ42 | Meta-temporal (CenTaur) | -0.52 | 0.009 |
| Aβ42 | Left meta-temporal SUVR | -0.54 | 0.006 |
| Aβ42 | Right meta-temporal SUVR | -0.45 | 0.028 |
| Aβ42 | Temporo-parietal (CenTaur) | -0.44 | 0.034 |
| Aβ42 | Left temporo-parietal SUVR | -0.44 | 0.032 |
| Aβ42 | Right temporo-parietal SUVR | -0.4 | 0.055 |
| Aβ42 | Global neocortex (CenTaur) | -0.47 | 0.020 |
| Aβ42 | Left hemisphere SUVR | -0.46 | 0.023 |
| Aβ42 | Right hemisphere SUVR | -0.46 | 0.024 |
| GFAP | Frontal cortex (CenTaur) | 0.12 | 0.588 |
| GFAP | Left frontal SUVR | 0.13 | 0.560 |
| GFAP | Right frontal SUVR | 0.1 | 0.627 |
| GFAP | Mesial temporal (CenTaur) | 0.33 | 0.121 |
| GFAP | Left mesial temporal SUVR | 0.32 | 0.132 |
| GFAP | Right mesial temporal SUVR | 0.32 | 0.126 |
| GFAP | Meta-temporal (CenTaur) | 0.45 | 0.027 |
| GFAP | Left meta-temporal SUVR | 0.46 | 0.022 |
| GFAP | Right meta-temporal SUVR | 0.39 | 0.063 |
| GFAP | Temporo-parietal (CenTaur) | 0.11 | 0.599 |
| GFAP | Left temporo-parietal SUVR | 0.17 | 0.433 |
| GFAP | Right temporo-parietal SUVR | 0.04 | 0.857 |
| GFAP | Global neocortex (CenTaur) | 0.19 | 0.362 |
| GFAP | Left hemisphere SUVR | 0.24 | 0.259 |
| GFAP | Right hemisphere SUVR | 0.13 | 0.541 |
| pTau217 | Frontal cortex (CenTaur) | 0.37 | 0.074 |
| pTau217 | Left frontal SUVR | 0.37 | 0.079 |
| pTau217 | Right frontal SUVR | 0.37 | 0.076 |
| pTau217 | Mesial temporal (CenTaur) | 0.57 | 0.004 |
| pTau217 | Left mesial temporal SUVR | 0.53 | 0.007 |
| pTau217 | Right mesial temporal SUVR | 0.58 | 0.003 |
| pTau217 | Meta-temporal (CenTaur) | 0.69 | <0.001 |
| pTau217 | Left meta-temporal SUVR | 0.68 | <0.001 |
| pTau217 | Right meta-temporal SUVR | 0.64 | 0.001 |
| pTau217 | Temporo-parietal (CenTaur) | 0.37 | 0.078 |
| pTau217 | Left temporo-parietal SUVR | 0.4 | 0.056 |
| pTau217 | Right temporo-parietal SUVR | 0.3 | 0.149 |
| pTau217 | Global neocortex (CenTaur) | 0.46 | 0.024 |
| pTau217 | Left hemisphere SUVR | 0.48 | 0.019 |
| pTau217 | Right hemisphere SUVR | 0.42 | 0.042 |
| pTau217/Aβ42 ratio | Frontal cortex (CenTaur) | 0.41 | 0.046 |
| pTau217/Aβ42 ratio | Left frontal SUVR | 0.39 | 0.062 |
| pTau217/Aβ42 ratio | Right frontal SUVR | 0.43 | 0.037 |
| pTau217/Aβ42 ratio | Mesial temporal (CenTaur) | 0.56 | 0.004 |
| pTau217/Aβ42 ratio | Left mesial temporal SUVR | 0.55 | 0.005 |
| pTau217/Aβ42 ratio | Right mesial temporal SUVR | 0.56 | 0.005 |
| pTau217/Aβ42 ratio | Meta-temporal (CenTaur) | 0.69 | <0.001 |
| pTau217/Aβ42 ratio | Left meta-temporal SUVR | 0.69 | <0.001 |
| pTau217/Aβ42 ratio | Right meta-temporal SUVR | 0.62 | 0.001 |
| pTau217/Aβ42 ratio | Temporo-parietal (CenTaur) | 0.41 | 0.049 |
| pTau217/Aβ42 ratio | Left temporo-parietal SUVR | 0.43 | 0.036 |
| pTau217/Aβ42 ratio | Right temporo-parietal SUVR | 0.35 | 0.098 |
| pTau217/Aβ42 ratio | Global neocortex (CenTaur) | 0.49 | 0.014 |
| pTau217/Aβ42 ratio | Left hemisphere SUVR | 0.5 | 0.012 |
| pTau217/Aβ42 ratio | Right hemisphere SUVR | 0.46 | 0.024 |

Partial correlation coefficients (age- and sex-adjusted) between plasma biomarker levels and tau PET SUVRs across Braak stage–related cortical regions in the cognitively unimpaired group.
*Abbreviations:* Aβ=amyloid beta; CU=cognitively unimpaired; Aβ42 = amyloid beta 42; GFAP = glial fibrillary acidic protein; pTau217 = phosphorylated tau at threonine 217; SUVR = standardized uptake value ratio

**Supplementary Table S12. Partial correlation coefficients between plasma biomarkers and regional tau PET SUVRs in the in the cognitively impaired (MCI+AD dementia) group (N=52)**

| Biomarker | Region | r | p |
| --- | --- | --- | --- |
| Aβ42 | Frontal cortex (CenTaur) | -0.16 | 0.258 |
| Aβ42 | Left frontal SUVR | -0.14 | 0.312 |
| Aβ42 | Right frontal SUVR | -0.17 | 0.228 |
| Aβ42 | Mesial temporal (CenTaur) | -0.15 | 0.296 |
| Aβ42 | Left mesial temporal SUVR | -0.15 | 0.305 |
| Aβ42 | Right mesial temporal SUVR | -0.15 | 0.298 |
| Aβ42 | Meta-temporal (CenTaur) | -0.19 | 0.182 |
| Aβ42 | Left meta-temporal SUVR | -0.17 | 0.216 |
| Aβ42 | Right meta-temporal SUVR | -0.19 | 0.173 |
| Aβ42 | Temporo-parietal (CenTaur) | -0.15 | 0.284 |
| Aβ42 | Left temporo-parietal SUVR | -0.12 | 0.391 |
| Aβ42 | Right temporo-parietal SUVR | -0.17 | 0.230 |
| Aβ42 | Global neocortex (CenTaur) | -0.17 | 0.231 |
| Aβ42 | Left hemisphere SUVR | -0.15 | 0.300 |
| Aβ42 | Right hemisphere SUVR | -0.18 | 0.194 |
| GFAP | Frontal cortex (CenTaur) | 0.31 | 0.026 |
| GFAP | Left frontal SUVR | 0.32 | 0.020 |
| GFAP | Right frontal SUVR | 0.28 | 0.047 |
| GFAP | Mesial temporal (CenTaur) | 0.4 | 0.003 |
| GFAP | Left mesial temporal SUVR | 0.38 | 0.005 |
| GFAP | Right mesial temporal SUVR | 0.41 | 0.002 |
| GFAP | Meta-temporal (CenTaur) | 0.36 | 0.009 |
| GFAP | Left meta-temporal SUVR | 0.36 | 0.009 |
| GFAP | Right meta-temporal SUVR | 0.33 | 0.018 |
| GFAP | Temporo-parietal (CenTaur) | 0.34 | 0.013 |
| GFAP | Left temporo-parietal SUVR | 0.38 | 0.006 |
| GFAP | Right temporo-parietal SUVR | 0.29 | 0.037 |
| GFAP | Global neocortex (CenTaur) | 0.35 | 0.010 |
| GFAP | Left hemisphere SUVR | 0.37 | 0.006 |
| GFAP | Right hemisphere SUVR | 0.32 | 0.023 |
| pTau217 | Frontal cortex (CenTaur) | 0.15 | 0.299 |
| pTau217 | Left frontal SUVR | 0.16 | 0.264 |
| pTau217 | Right frontal SUVR | 0.13 | 0.370 |
| pTau217 | Mesial temporal (CenTaur) | 0.17 | 0.218 |
| pTau217 | Left mesial temporal SUVR | 0.15 | 0.279 |
| pTau217 | Right mesial temporal SUVR | 0.19 | 0.183 |
| pTau217 | Meta-temporal (CenTaur) | 0.12 | 0.411 |
| pTau217 | Left meta-temporal SUVR | 0.12 | 0.408 |
| pTau217 | Right meta-temporal SUVR | 0.11 | 0.455 |
| pTau217 | Temporo-parietal (CenTaur) | 0.16 | 0.267 |
| pTau217 | Left temporo-parietal SUVR | 0.17 | 0.234 |
| pTau217 | Right temporo-parietal SUVR | 0.14 | 0.325 |
| pTau217 | Global neocortex (CenTaur) | 0.16 | 0.261 |
| pTau217 | Left hemisphere SUVR | 0.17 | 0.238 |
| pTau217 | Right hemisphere SUVR | 0.14 | 0.313 |
| pTau217/Aβ42 ratio | Frontal cortex (CenTaur) | 0.44 | 0.001 |
| pTau217/Aβ42 ratio | Left frontal SUVR | 0.43 | 0.001 |
| pTau217/Aβ42 ratio | Right frontal SUVR | 0.42 | 0.002 |
| pTau217/Aβ42 ratio | Mesial temporal (CenTaur) | 0.43 | 0.001 |
| pTau217/Aβ42 ratio | Left mesial temporal SUVR | 0.4 | 0.003 |
| pTau217/Aβ42 ratio | Right mesial temporal SUVR | 0.44 | 0.001 |
| pTau217/Aβ42 ratio | Meta-temporal (CenTaur) | 0.43 | 0.001 |
| pTau217/Aβ42 ratio | Left meta-temporal SUVR | 0.42 | 0.002 |
| pTau217/Aβ42 ratio | Right meta-temporal SUVR | 0.42 | 0.002 |
| pTau217/Aβ42 ratio | Temporo-parietal (CenTaur) | 0.45 | 0.001 |
| pTau217/Aβ42 ratio | Left temporo-parietal SUVR | 0.45 | 0.001 |
| pTau217/Aβ42 ratio | Right temporo-parietal SUVR | 0.43 | 0.001 |
| pTau217/Aβ42 ratio | Global neocortex (CenTaur) | 0.47 | <0.001 |
| pTau217/Aβ42 ratio | Left hemisphere SUVR | 0.47 | <0.001 |
| pTau217/Aβ42 ratio | Right hemisphere SUVR | 0.46 | 0.001 |

Partial correlation coefficients (age- and sex-adjusted) between plasma biomarker levels and tau PET SUVRs across Braak stage–related cortical regions in the cognitively impaired group.
*Abbreviations:* Aβ42 = amyloid beta 42; GFAP = glial fibrillary acidic protein; pTau217 = phosphorylated tau at threonine 217; SUVR = standardized uptake value ratio

**Supplementary figures**

**Supplementary Figure S1. ROC curve comparison for a) biomarker-only vs. b) biomarker+APOE4 models in cognitively unimpaired group (Aβ−CU and Aβ+CU) (N=94)**


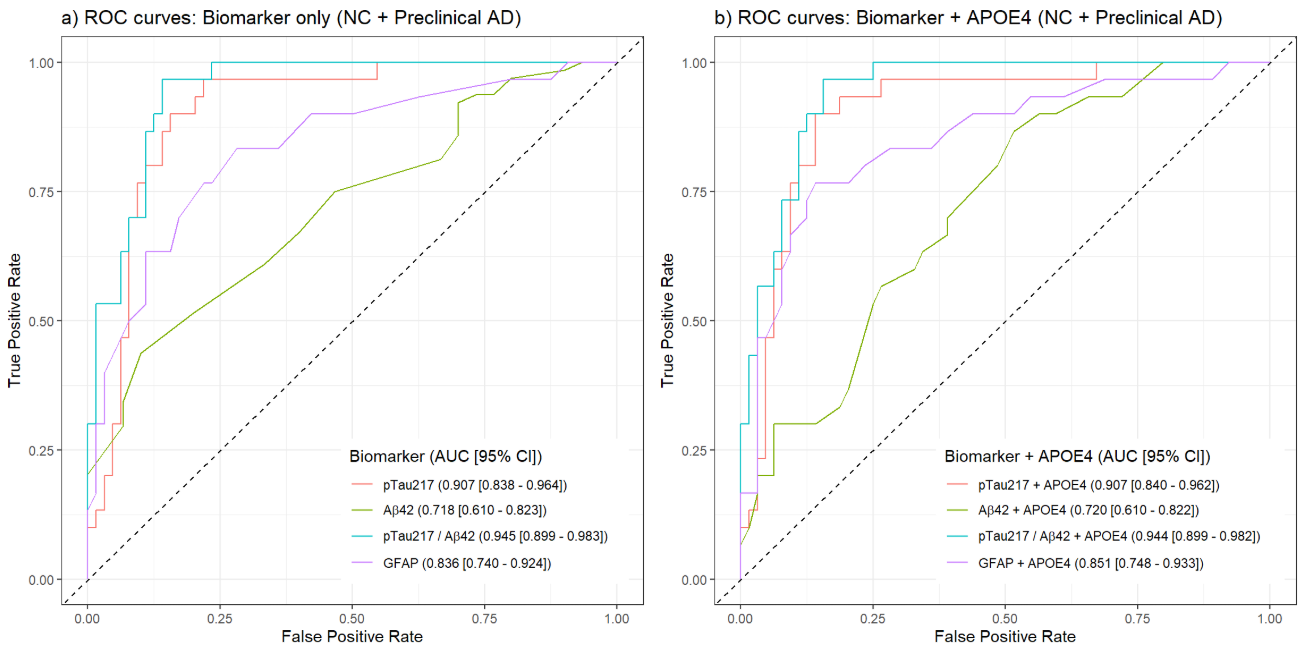


CL value >20 was used to define amyloid positivity

***Abbreviations:*** Aβ=amyloid beta; CU=cognitively unimpaired; pTau217 = phosphorylated tau at threonine 217; Aβ42 = amyloid-beta 42; GFAP = glial fibrillary acidic protein; CL = Centiloid; APOE4 = apolipoprotein E ε4 allele.

**Supplementary Figure S2. ROC curve comparison for a) biomarker-only vs. b) biomarker+APOE4 models in cognitively impaired (MCI and AD dementia) group(N=144)**


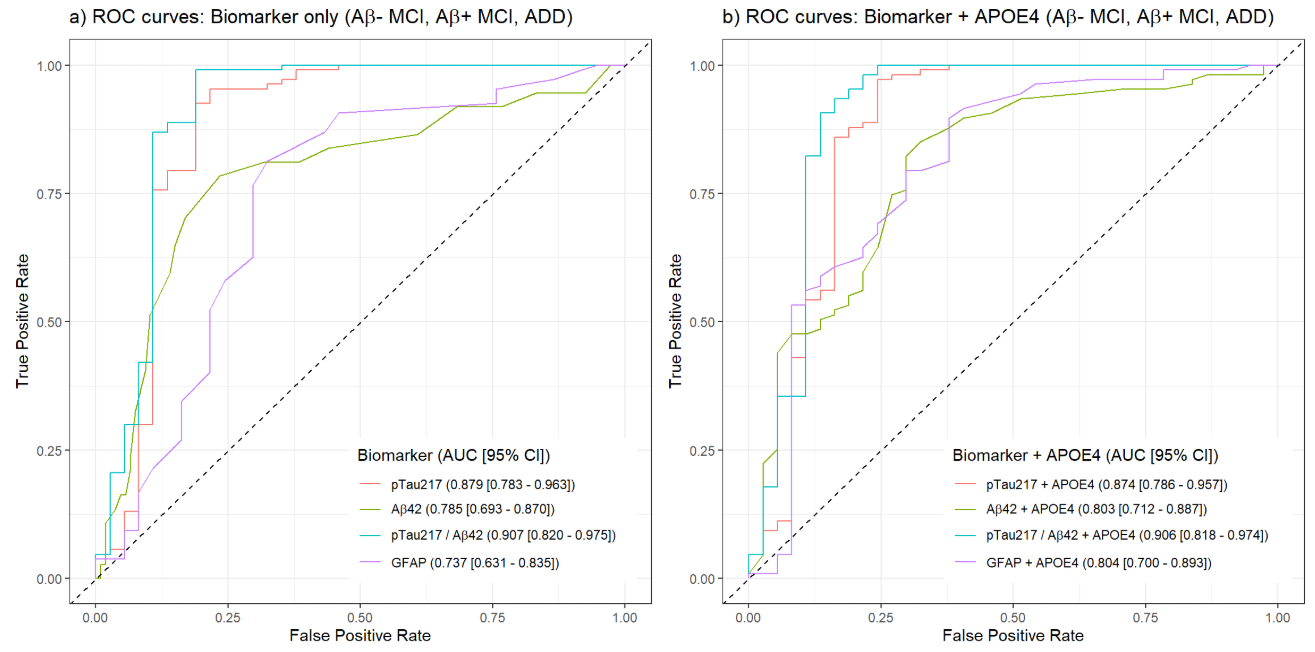


CL value >20 was used to define amyloid positivity

***Abbreviations:*** MCI=mild cognitive impairment, ADD=Alzheimer’s disease dementia; pTau217 = phosphorylated tau at threonine 217; Aβ42 = amyloid-beta 42; GFAP = glial fibrillary acidic protein; CL = Centiloid; APOE4 = apolipoprotein E ε4 allele.

**Supplementary Figure S3. Partial correlations between plasma biomarkers and tau PET SUVRs in the cognitively unimpaired group (Aβ−CU and Aβ+CU) (N=24)**

**
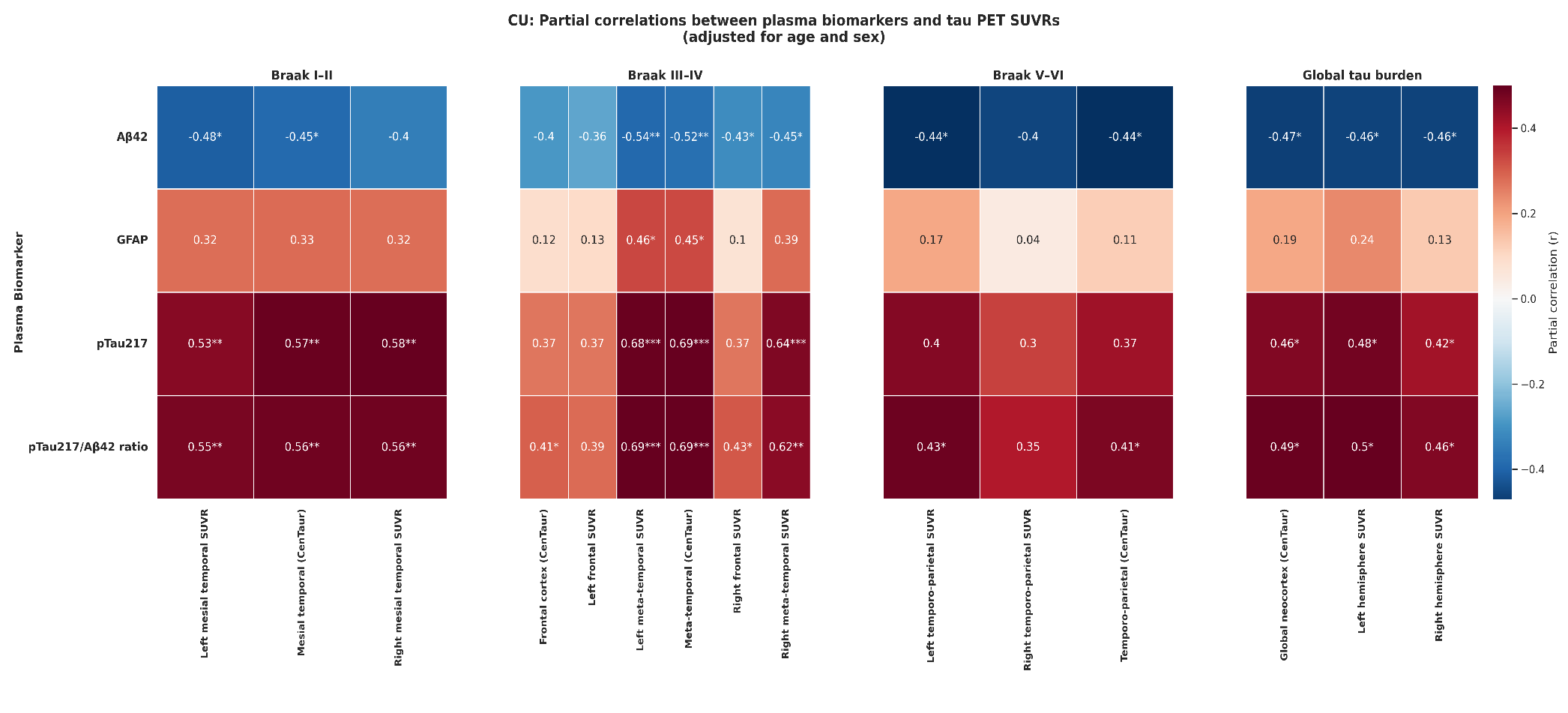
**

Partial correlation coefficients between plasma biomarkers (Aβ42, GFAP, pTau217, and pTau217/Aβ42 ratio) and regional tau PET SUVRs in the cognitively unimpaired group, adjusted for age and sex using a residual-based method. Regional tau PET variables were grouped according to Braak stage–related cortical topography, with four subplot panels representing Braak I–II, III–IV, V–VI, and global tau burden. Significant correlations are annotated (*p < .05; **p < .01; ***p < .001), and color represents the strength and direction of correlation (r).

*Abbreviations:* Aβ=amyloid beta; CU=cognitively unimpaired; PET=positron emission tomography; SUVR=standardized uptake value ratio; Aβ42 = amyloid beta 42; GFAP = glial fibrillary acidic protein; pTau217 = phosphorylated tau at threonine 217

**Supplementary Figure S4. Partial correlations between plasma biomarkers and tau PET SUVRs in the cognitively impaired (MCI+AD dementia) group (N=52)**


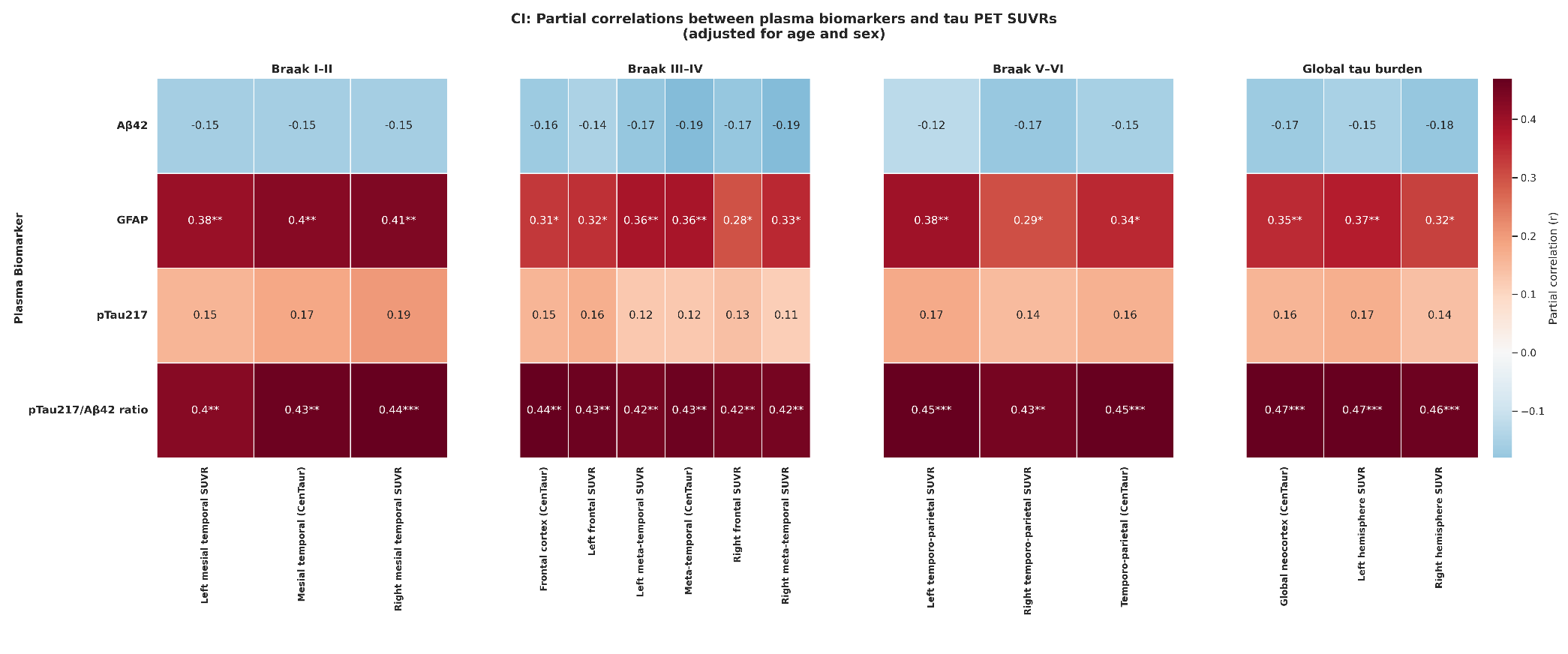


Partial correlations between plasma biomarkers and regional tau PET SUVRs in the cognitively impaired (CI) group, following adjustment for age and sex. Regions were categorized by Braak-stage–aligned topography. Heatmaps depict associations for Braak I–II, III–IV, V–VI, and global neocortical tau burden. Statistical significance is indicated (*p < .05; **p < .01; ***p < .001), with redder shades indicating stronger positive associations and bluer shades indicating negative associations.

*Abbreviations:* PET=positron emission tomography; SUVR=standardized uptake value ratio; MCI=mild cognitive impairment; AD=Alzheimer’s disease; Aβ42 = amyloid beta 42; GFAP = glial fibrillary acidic protein; pTau217 = phosphorylated tau at threonine 217

**Supplementary References**

1. Lee JH, Lee KU, Lee DY, et al. Development of the Korean version of the Consortium to Establish a Registry for Alzheimer's Disease Assessment Packet (CERAD-K): clinical and neuropsychological assessment batteries. *J Gerontol B Psychol Sci Soc Sci*. 2002;57(1):P47-53.

2. Jack CR Jr, Andrews JS, Beach TG, Buracchio T, Dunn B, Graf A, Hansson O, et al. Revised criteria for diagnosis and staging of Alzheimer's disease: Alzheimer's Association Workgroup. Alzheimers Dement. 2024;20(8):5143–5169. doi:10.1002/alz.13859.

3. Morris JC. Clinical dementia rating: a reliable and valid diagnostic and staging measure for dementia of the Alzheimer type. *Int Psychogeriatr*. 1997;9 Suppl 1:173-176; discussion 177-178.

4. Petersen RC, Caracciolo B, Brayne C, Gauthier S, Jelic V, Fratiglioni L. Mild cognitive impairment: a concept in evolution. *J Intern Med*. 2014;275(3):214-228.
